# Thermal bottlenecks constrain early-life survival of native fish in regulated rivers

**DOI:** 10.1126/sciadv.aeg3586

**Published:** 2026-08-21

**Authors:** Kohma Arai, Rachael E. Ryan, Miles Daniels, Malte Willmes, George E. Whitman, Larisa M. Thacher, Nozomi Matsuda, Elizabeth A. Bell, Kevin D. McKeegan, Carson A. Jeffres, Rachel C. Johnson

**Affiliations:** ^1^ Center for Watershed Sciences, University of California Davis, Davis, CA, USA.; ^2^ Fisheries Collaborative Program, University of California Santa Cruz, Santa Cruz, CA, USA.; ^3^ Southwest Fisheries Science Center, NOAA Fisheries, Santa Cruz, CA, USA.; ^4^ Norwegian Institute for Nature Research, PO Box 5685, 7485, Torgarden, Trondheim, Norway.; ^5^ Department of Earth, Planetary and Space Sciences, University of California Los Angeles, Los Angeles, CA, USA.

## Abstract

Climate change is reshaping thermal regimes worldwide, threatening species whose early development depends on narrow temperature ranges. Yet directly linking warming to reproductive failure in wild populations remains intractable, particularly in aquatic ecosystems. We developed an empirical framework integrating stable isotope thermometry of archival tissues, landscape-scale temperature modeling, and field surveys to identify when and where successful reproduction occurs. Applying this approach to endangered winter-run Chinook salmon confined below a large dam, we revealed a narrow “critical thermal window” during early development. Surviving juveniles experienced temperatures below 12.5°C within ∼15 days of fertilization, and modest warming during this period sharply reduced survival. In warm years, dam-released cold-water overlapped only briefly with this window, restricting successful reproduction to a small fraction of the total spawning season. These results show how climate warming and river regulation interact to constrain population persistence and provide a framework to guide research on assessing climate vulnerability in temperature-sensitive species.

## INTRODUCTION

Climate change and habitat alteration are rapidly transforming rivers, lakes, and streams worldwide, intensifying thermal extremes and altering flow regimes (*1*). Freshwater ecosystems, while disproportionately biodiverse compared to marine and terrestrial systems, are also among the most vulnerable to climate change, with many species facing range contractions, phenological shifts, or local extinctions (*2*, *3*). For cold-blooded aquatic animals, temperature is one of several interacting factors governing survival, growth, and reproduction (*4*). Yet, unlike many terrestrial or marine species that can shift poleward or upslope to track suitable climates (*5*, *6*), freshwater-dependent organisms are constrained by basin boundaries, fragmentation, and limited connectivity, severely limiting their capacity to track shifting thermal niches. These constraints make freshwater-dependent biota especially vulnerable to warming, particularly where river regulation and water management further modify thermal regimes (*7*).

A central challenge in predicting climate impacts on freshwater biodiversity is linking warming conditions to population-level reproductive failure in the wild (*8*). Early developmental stages are often the most temperature sensitive (*9*, *10*), yet mortality during these stages is rarely observed directly, especially in aquatic systems where dead individuals go undetected (*11*, *12*). Consequently, it remains difficult to determine when, where, and under what environmental conditions mortality occurs in the wild, particularly when individuals cannot be tracked continuously throughout development (*13*, *14*). This limitation has hindered efforts to empirically identify critical thermal thresholds, vulnerable developmental windows, and spatial “mortality hotspots” that ultimately govern population persistence under climate change.

Experimental and mechanistic studies across fishes and other aquatic ectotherms demonstrate that mortality risk during early development can be concentrated within narrow, discrete windows of heightened sensitivity (*15*–*17*). In salmonids, for example, one such window occurs during gastrulation, when thermal disruption of gene expression and developmental stability can lead to immediate or delayed mortality (*17*). A second window arises immediately prior to hatch, when rapidly increasing metabolic demand can exceed the oxygen that diffuses through the egg membrane (*15*, *16*, *18*). These studies suggest that even modest warming, if it coincides with such vulnerable periods, can have disproportionate consequences for recruitment. However, whether and how these critical thermal windows translate into selective mortality in the wild, where temperature varies across space and time and interacts with habitat constraints, remains largely unresolved.

These challenges are especially acute in regulated river systems, where dams alter flow regimes, constrain access to historical habitats, and impose strong spatial and temporal structure on thermal conditions (*7*). In such systems, the persistence of temperature-sensitive species can depend not only on absolute temperature limits, but also on the timing and duration of suitable thermal conditions relative to early developmental stages. Understanding how climate warming interacts with river regulation to shape early-life survival therefore requires approaches that can reconstruct fine-scale thermal exposure and survival outcomes directly from individuals in natural environments.

**Fig. 1.**
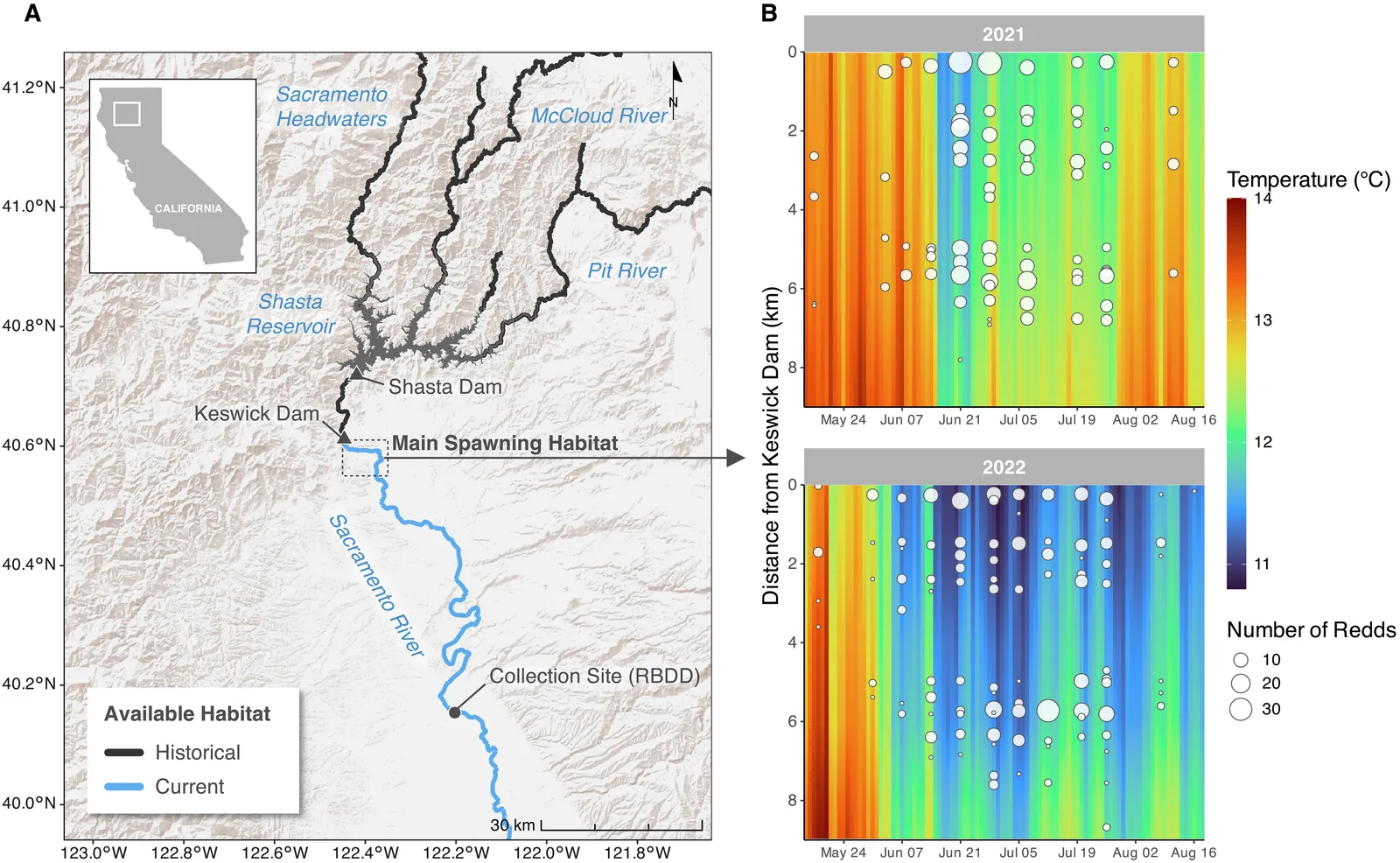
Habitat range and spawning distribution of Sacramento River winter-run Chinook salmon. (**A**) Historical (black) and current (blue) available habitats of Central Valley winter-run Chinook salmon in the Sacramento River, showing major dams (triangles), main spawning habitat (dashed rectangle), and the juvenile collection site at the Red Bluff Diversion Dam (solid circle, RBDD). (**B**) Spatial and temporal distribution of salmon gravel nests (redds) during the main spawning season in 2021 and 2022, from aerial surveys, overlaid on 2D water temperature contours from the River Assessment for Forecasting Temperature (RAFT) model.

The endangered Sacramento River winter-run Chinook salmon (*Oncorhynchus tshawytscha*) in California’s Central Valley provides a rare and powerful test case for addressing this problem (*19*). This population exhibits a unique life-history strategy of summer spawning (June–August) that evolved in cold, spring-fed mountain streams (*20*). Adults deposit eggs in gravel nests (“redds”), where embryos develop for several weeks before hatching as alevins, which remain buried until emerging as fry approximately one month later (*20*). However, large dams now block access to their historical spawning grounds, confining the entire population to a single low-elevation reach below Keswick Dam and making successful reproduction entirely dependent on cold-water releases from Shasta Reservoir, whose availability is constrained by both meteorological conditions and competing water demands (Fig. 1A). This cold water is a limited and increasingly contested resource during droughts (*21*). When reservoir storage is low, downstream river temperatures can rise, causing high mortality during thermally sensitive stages of early development (*15*, *16*) (Fig. 1B). Occupying the southernmost edge of the species’ range and persisting under extreme habitat restriction, Sacramento River winter-run Chinook salmon represent a model species for examining how climate warming, water management, and habitat restriction interact to shape reproductive success in freshwater ecosystems.

Despite their ecological, cultural, and management importance, little is known about when and where most early-life mortality occurs in this system. Spawning surveys document where and when eggs are deposited, but they do not reveal which spawning events ultimately produce surviving juveniles. Conversely, juvenile migrants sampled downstream represent only the survivors of early development, with no direct information on the thermal conditions experienced by individuals that perished. Bridging this gap requires approaches that can link individual survival histories to spatially explicit thermal environments.

Here, we address this challenge by integrating three complementary data sources: otolith microstructure and stable isotope thermometry, extensive aerial redd surveys, and landscape-scale temperature models. Otoliths, paired calcium carbonate structures in the inner ear of fishes, are natural archives that record both daily growth increments (age) and the chemical signature of ambient water (*22*). Stable oxygen isotope ratios (δ^18^O) in otoliths also vary predictably with water temperature (*23*–*25*), enabling reconstruction of individual thermal histories when paired with environmental isotope data (*26*, *27*). By linking thermal exposures of surviving juveniles to population-level spawning activity and spatially explicit river temperatures, we were able to (i) geolocate natal origins of juveniles that survived early mortality and were collected at downstream traps, (ii) identify where and under what conditions reproduction failed, and (iii) pinpoint the developmental window most sensitive to thermal stress.

We apply this empirical framework to two contrasting years, 2021 (warm) and 2022 (cool), which provide a natural experiment in thermal constraint. Here, “warm” and “cool” refer to differences in river water temperature regimes experienced during spawning and early incubation, driven by contrasting cold-water availability and reservoir operations. In 2021, limited cold-water storage in Shasta Reservoir restricted suitable spawning temperatures (<12°C) (*15*) to a brief period (Fig. 1B), whereas in 2022, reservoir operations maintained suitable thermal conditions in the 8 km downstream of Keswick Dam for much of the spawning season. These contrasting conditions allow us to evaluate how the alignment or mismatch between thermal regimes and early developmental windows governs reproductive success. More broadly, our approach provides an integrative framework for diagnosing climate vulnerability, identifying thermal bottlenecks, and informing management strategies for temperature-sensitive species.

## RESULTS

### Reproductive success was temporally constrained in the warm year

**Fig. 2.**
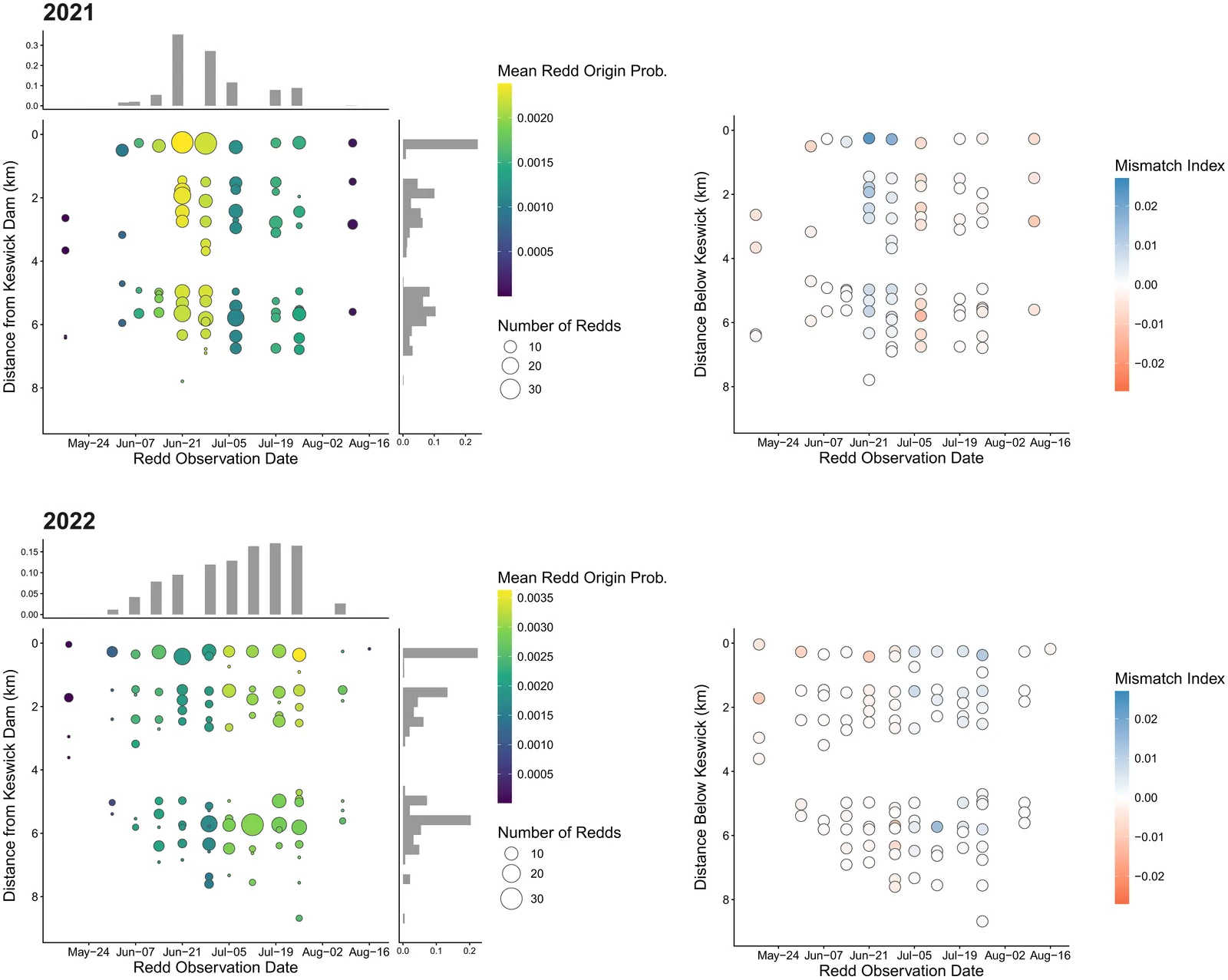
Spatial and temporal patterns of successful redds for winter-run Chinook salmon. Population-level reproductive success (left) and proportional mismatch (right) for juvenile winter-run Chinook salmon survivors collected at the downstream Red Bluff Diversion Dam in 2021 (top row) and 2022 (bottom row). Mean redd origin probabilities (i.e., survival) were estimated as the product of individual hatch-date and temperature probabilities, averaged across individuals within each year. Marginal histograms show the number of redds weighted by mean redd origin probability, highlighting the timing and location of successful redds. Note that the color scale for mean redd origin probability differs between 2021 and 2022 to aid visualization. Proportional mismatch panels show the difference between expected redd proportions and observed survival probabilities, where values near zero (white) indicate close agreement, and positive (blue) or negative (red) deviations indicate disproportionate survival. See fig. S4 for distribution of redd origin probabilities projected across the Sacramento River basin.

Reproductive success in the warm year (2021) was restricted to a narrow spawning window (Fig. 2). By reconstructing hatch dates and early-life temperature from otoliths (2021: *n* = 40; 2022: *n* = 35), and linking these with high-resolution river temperature models, we estimated the likely spawning location (“redd origin”) of each surviving juvenile Chinook salmon collected downstream (fig. S1). In 2021, the highest mean redd origin probabilities of survivors were concentrated in redds observed between the weeks of June 21 and June 28, but spanning a relatively broad spatial range (Fig. 2, top left). Redds outside this temporal window disproportionately produced fewer survivors than expected based on redd counts alone (Fig. 2, top right). The narrow temporal concentration of successful redds was resolved with relatively high precision because hatch-date reconstruction provided strong temporal constraint on redd assignment.

In contrast, during the cooler year of 2022, despite a similar overall spawning period, survivors tended to originate later in the season and over a broader temporal window, from mid-June to late July. However, successful redds were more spatially concentrated, with higher probabilities clustered within 2 km downstream of Keswick Dam (Fig. 2, bottom left). Redds observed in this area and during early to late July disproportionately produced survivors, whereas redds deposited earlier in the season contributed fewer survivors than expected (Fig. 2, bottom right).

Underlying otolith hatch-date and early-life temperature reconstruction data of juvenile survivors in 2021 and 2022 are provided in text S1.

### Early incubation temperatures strongly predict survival

**Fig. 3.**
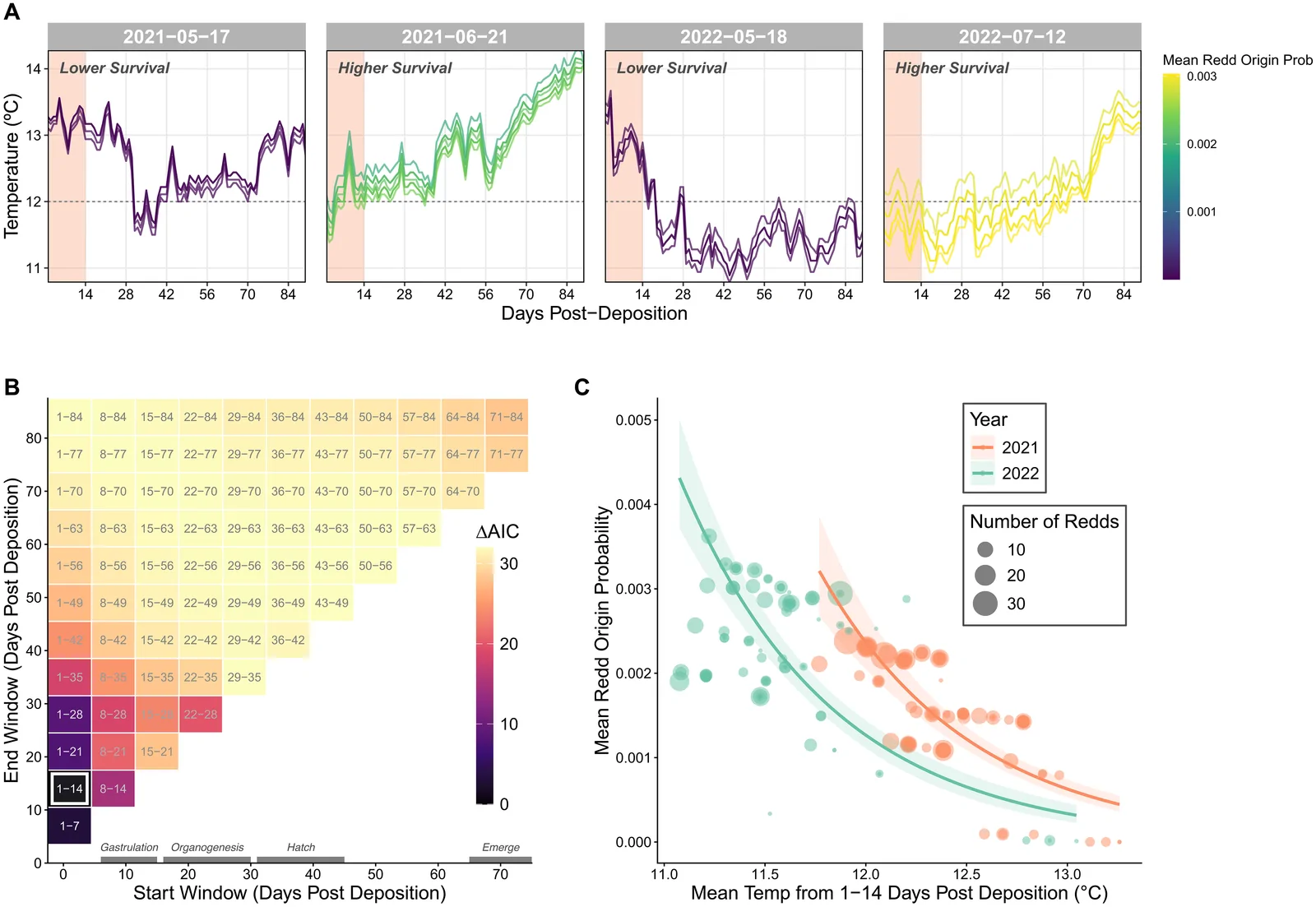
Critical thermal window for early-life survival in winter-run Chinook salmon. (**A**) Example daily temperature profiles estimated from the RAFT (River Assessment for Forecasting Temperature) model for observed redd groups (redds sharing the same observation date and spatial location), colored by mean redd origin probability (i.e., inferred survival). Facets show representative redd groups spanning both relatively high and low inferred survival outcomes across deposition dates and years. Panels are separated by redd deposition date. The pink shaded area marks the identified critical thermal window (1–14 days post-deposition), and the dashed horizontal line indicates the 12°C reference threshold. Modeled temperature profiles for all observed redds are shown in fig. S5. (**B**) Heatmap of ΔAIC (difference in Akaike Information Criterion) values from the moving window generalized linear model (GLM) analysis. For each start–end window combination, mean temperatures were calculated from RAFT-modeled redd temperature histories and related to inferred survival (mean redd-origin probability). Each grid cell represents a specific start-end window combination, with the corresponding ΔAIC value indicating model fit. The best-fitting model (ΔAIC = 0) identified a critical window of 1–14 days post-redd deposition highlighted with a white box. Developmental stages and their approximate durations are shown along the x-axis. (**C**) Relationship between the mean redd temperature during the critical window (1–14 days post-deposition, identified as the best-fitting model) and survival in 2021 and 2022. Points represent observed redd groups with the point size proportional to the number of redds in each group. Fitted lines represent model-predicted marginal effects of mean temperature on survival from the gamma GLM, holding redd count constant at the year-specific median. Shaded areas represent 95% confidence intervals around the marginal effect estimates.

Survival was most strongly influenced by temperatures experienced during the first two weeks of incubation. Using a moving window generalized linear model that systematically evaluated modeled redd temperature histories in relation to inferred survival, we identified days 1–14 as the “critical thermal window” most predictive of survival (Fig. 3B). Temperature windows spanning days 1–28 also showed strong support, whereas later incubation periods had substantially weaker associations, indicating that early development is the most temperature-sensitive phase. This early critical window was consistent with the visual inspection of modeled daily temperature profiles, which showed that redds with higher inferred survival consistently experienced cooler temperatures during the first two weeks after deposition (Fig. 3A).

For both 2021 and 2022, survival probability declined sharply with increasing temperatures during this critical period (Gamma GLM, log link; β = −1.33, SE = 0.11, *PP* < 0.001; Fig. 3C), equivalent to an approximately 73% decrease in survival per 1°C increase in mean temperature. Redds that experienced high mean temperatures (>12.5°C) during this early period showed a ∼3-fold reduction (0.0007 vs. 0.0022) in mean redd origin probabilities compared to cooler conditions. Importantly, however, after accounting for mean temperature during the critical window and redd abundance, baseline mean redd origin probability remained significantly lower in 2022 than in 2021 (year effect: β = −0.61, SE = 0.12, *PP* < 0.001), indicating additional sources of mortality beyond temperature alone.

## DISCUSSION

Early-life survival often governs population persistence, yet the timing and mechanisms of mortality in wild populations remain difficult to resolve. Here, we show that survival in a temperature-sensitive freshwater species is largely determined within a narrow early developmental window, revealing a physiological bottleneck through which climate warming and river regulation can push a species towards collapse. More broadly, our results demonstrate how warming temperatures, constrained habitats, and managed flow regimes interact to generate selective early-life mortality, with consequences that extend well beyond this system to regulated rivers worldwide.

Fine-scale geolocation of natal origins revealed that survival was strongly constrained in time by warm conditions, with successful redds in 2021 concentrated within a narrow spawning window of just a few cold weeks (<12.5°C) in June (Fig. 2; text S2.1). This pattern largely reflects reservoir operations: in 2021, the Shasta cold-water pool was depleted early, limiting thermal suitability to a brief period that coincided with peak spawning activity, whereas in 2022, cooler conditions extended into late July. These findings highlight how the overlap, or mismatch, between managed cold-water releases and spawning phenology determines which redds produce recruits. As a result, even when redds are broadly distributed throughout the spawning season, only a fraction contributes to the next generation in warm years.

This temporal constriction can substantially reduce effective population size (number of individuals that successfully contribute genes to the next generation) relative to census size (number of total spawners present). Such reductions can erode genetic diversity, increase the effects of genetic drift and inbreeding, and diminish the capacity of populations to adapt to environmental change (*28*, *29*). Consequently, populations with low effective population sizes are more vulnerable to demographic and environmental stochasticity, less resilient to climate extremes, and face elevated extinction risk (*30*). These concerns are particularly acute for endangered species, where census abundance is often used as a proxy for the more difficult-to-measure effective population size, potentially obscuring critically low effective population sizes and the accompanying loss of evolutionary and recovery potential (*31*). By identifying the spatial and temporal components of the spawning population that successfully contribute recruits, our otolith-based framework provides a means of assessing how environmental conditions influence this key viability metric.

We identified a “critical thermal window,” a period during early embryonic development when survival is highly sensitive to temperature. Such narrow developmental windows have been proposed for many ectotherms but are rarely demonstrated empirically in wild populations. Moving window models consistently indicated this early phase as the strongest predictor of survival, with the best-fitting model spanning 1–14 days post-fertilization (Fig. 3; text S2.2). Survival during this period exhibited a steep, non-linear decline with increasing temperature, with relatively small temperature increases resulting in drastic losses. Within this period, mean temperatures above ∼12.5°C were associated with extremely low survival, whereas individuals that survived beyond this stage often endured much warmer temperatures later in development (>14°C during post-hatch alevin stages).

Previous laboratory and modeling studies have identified two distinct early-stage thermal sensitivity windows in salmonids: an early one during gastrulation, when elevated temperatures disrupt gene expression and development (*17*), and a later one immediately before hatch, when rapidly rising metabolic demand exceeds oxygen diffusion through the egg membrane (*16*, *18*). Gastrulation establishes the embryo’s longitudinal axis and the foundational structures for subsequent organogenesis, which appears less temperature sensitive (*17*). Elevated temperatures during gastrulation can cause immediate mortality, and disruptions at this stage may carry over to increase mortality during hatching (*17*). Our empirical analyses provided strongest support for an early window spanning 1–14 days post-fertilization, with slightly weaker support for broader windows extending up to 28 days. Results were highly consistent when using accumulated temperature units (ATU) instead of days post-deposition, with the 0–140 ATU window selected as the strongest period of temperature sensitivity (see text S3). This period aligns closely with the gastrulation-related sensitivity [roughly 50–125 accumulated temperature units (*17*)], while not excluding the possibility that later pre-hatch processes also contribute to mortality. Although alternative mechanisms such as failed fertilization or reduced gamete viability cannot be completely excluded, the strong correspondence between the inferred critical window and experimentally identified embryonic thermal sensitivity periods suggests that temperature-dependent mortality during early development was the more likely driver of the observed survival patterns. Because our critical window identification relied on correlational models rather than direct physiological measurements, this mechanistic interpretation should be viewed cautiously. Nevertheless, the close agreement between our field-based reconstructions and experimental studies provides rare empirical evidence that thermal sensitivity during early development can strongly influence survival in natural populations.

Together, these results show that the temporal constriction of successful redds in warm years reflects a physiological bottleneck during early incubation. Under extreme drought, this combined effect of high temperatures and early-life sensitivity can translate into near-complete cohort failure, as observed during 2014 and 2015 (*32*). From a management perspective, this means cold-water strategies cannot rely on absolute temperature thresholds alone. The timing and duration of favorable thermal conditions are as critical as the temperature magnitude itself, and even short mismatches between spawning phenology and reservoir operations that govern river temperature regimes can trigger drastic losses in early-life survival and recruitment. Importantly, once fish survive this narrow early window, they appear capable of tolerating higher temperatures later in development, potentially offering greater flexibility for water operations later in the season. However, this does not imply an absence of thermal constraints during later developmental stages, as upper thermal limits and other temperature-related stressors remain important throughout incubation and rearing (*33*).

At the same time, caution is warranted when interpreting these results within an optimization framework. Broad spawning distributions in salmon are widely thought to function as bet-hedging strategies that spread reproductive risk across variable environmental conditions and contribute to portfolio effects that stabilize populations through time (*34*, *35*). Management actions that disproportionately favor a narrow portion of the spawning season may increase short-term survival under some conditions but could inadvertently reduce temporal diversity and resilience to future environmental variability. Accordingly, conservation strategies should seek not only to maximize survival during identified critical windows but also to maintain the life-history diversity that contributes to long-term population persistence.

Looking forward, climate change will likely further constrain the effectiveness of reservoir operations: projections indicate that Shasta Reservoir may be unable to maintain sufficient cold-pool volume throughout early incubation for winter-run Chinook salmon, even under extreme reservoir release reductions (*36*). These findings underscore the urgency of developing complementary strategies beyond temperature and flow management, including reestablishing independent populations in historical habitats (e.g., Battle Creek (*37*)) and above dams [e.g., McCloud River (*38*)], where more natural temperature regimes may provide long-term refugia for these species that evolved and were shaped by these habitats. Such efforts are particularly critical for winter-run Chinook salmon, whose entire spawning population is currently confined to a single river reach below Keswick Dam outside of their historical range. Restoring access to historical habitats would help spread the risk of cohort failure across multiple environments, fostering portfolio effects that enhance population resilience to future perturbations (*34*, *39*).

The strong temperature dependence of early survival was conserved across years; however, baseline survival was lower in 2022 even after accounting for thermal exposure during the critical window (Fig. 3C). Notably, although thermally suitable conditions (<12.5°C) persisted throughout June and July 2022 across the full ∼8 km spawning reach, successful redds were largely confined to the upper ∼4 km downstream of Keswick Dam (Fig. 2). This pronounced year effect indicates that additional sources of early-life mortality may have operated in 2022, consistent with the exceptionally low egg-to-fry survival observed that year despite broadly favorable thermal conditions (*40*). Potential contributors include record-low summer discharge in 2022, which may have constrained habitat quality or dissolved oxygen availability (*18*, *41*), as well as widespread mortality due to thiamine deficiency documented in recent years (*42*). These findings indicate that favorable temperatures during early incubation are a necessary but not sufficient condition for successful recruitment.

Our findings also carry broader implications for freshwater biodiversity in regulated rivers worldwide. Many aquatic species, including amphibians, macroinvertebrates, and fishes, have specific developmental stages that are highly sensitive to temperature dynamics (*9*, *43*–*45*), meaning that short-lived thermal bottlenecks can disproportionately shape offspring survival. In systems where climate warming interacts with reservoir operations, these physiological constraints may create severe early-life bottlenecks, disproportionately reducing recruitment even when spawning activity remains high. The winter-run Chinook salmon serves as a critical early-warning case study: its restricted habitat and reliance on managed cold-water releases foreshadow how climate change, water regulation, and habitat constraints may shape the fate of other temperature-sensitive aquatic organisms inhabiting regulated rivers at the edge of their thermal limits (*2*, *46*). More broadly, our results emphasize that effective conservation strategies must consider not only absolute temperature thresholds but also the fine-scale timing and duration of environmental flows. By highlighting how survival bottlenecks emerge from the interplay of physiology, climate, and water management, this work offers a general framework for understanding climate vulnerability across diverse freshwater taxa and systems.

A key contribution of this study is the integrative framework used to diagnose selective mortality in the wild. By combining otolith microstructure, stable isotope thermometry, high-resolution temperature models, and extensive redd surveys, we were able to reconstruct both the fine-scale timing and spatial origin of survivors while also interpreting these outcomes within the broader environmental background of reservoir operations. By comparing the reconstructed dates and locations of successful spawners in relation to the broader patterns of spawning activity and modeled temperatures, this design overcomes a major limitation of conventional tagging and otolith-based approaches, which typically capture only the conditions experienced by fish that survive. Importantly, independent genetic assignments showed generally consistent spatiotemporal patterns with the otolith-informed reconstructions, providing support for the robustness of the framework (see text S4). Because otoliths (and other archival tissues) are routinely collected for stock assessments, elements of this framework may be extendable to retrospective analyses of long-term environmental change and population-level responses in other systems. Together, these strengths provide an empirically grounded link between individual developmental histories and population survival patterns, which is an urgently needed capability for understanding climate vulnerabilities in temperature-sensitive species.

Despite the strengths of this framework, several limitations warrant consideration. First, retrospective reconstructions inherently rely on survivors, meaning that the precise timing and magnitude of mortality cannot be directly observed. Accordingly, selective mortality in this study was inferred indirectly through the underrepresentation of survivors associated with particular environmental conditions, rather than through direct observation of mortality events, which are rarely observed in natural systems. Additionally, while moving window models consistently highlighted early incubation as the critical thermal window, this was based on correlational analyses that identify the window most predictive of survival outcomes, rather than direct physiological inference. Moreover, we could not directly reconstruct temperatures during early incubation itself due to isotopic maternal overprinting (see text S5). Rather, otolith isotopic data provided temperatures immediately prior to emergence for redd geolocation, and earlier incubation conditions were inferred from modeled temperatures.

Second, the accuracy of natal geolocation depends on the resolution of otolith microstructure, isotope data, temperature models, and redd surveys. In this system, hatch dates spanned several months, making hatch-date reconstruction a highly informative constraint on redd assignment and enabling precise temporal inference of successful spawning periods (fig. S1A). In contrast, relatively weak spatial temperature gradients limited the spatial resolution of redd-origin assignments (fig. S1B). This limitation was compounded by uncertainty in otolith-based temperature reconstructions arising from analytical precision, uncertainty in the otolith thermometry equation, variation in water δ^18^O values, and environmental temperature model uncertainty. Combined uncertainty associated with individual temperature reconstructions was on the order of several degrees Celsius, limiting confidence in fine-scale inference of absolute temperatures for individual fish (fig. S1E). Accordingly, reconstructed temperatures should be interpreted probabilistically rather than as precise point estimates. Importantly, inference in this framework emerges from the combined spatial and temporal structure across many individuals, rather than from precise geolocation of individual fish. Otolith edge-validation analyses comparing otolith-derived temperatures with the observed river temperatures on the date of fish capture showed moderate explanatory power (adjusted R^2^ = 0.69) and substantial individual-level variability, but did not indicate strong systematic bias in reconstructed temperatures (text S6). To ensure conclusions did not overstate the level of certainty provided by the data, all identifiable sources of uncertainty were explicitly propagated into the redd-origin assignment framework (see “Redd origin probability model” in Materials and Methods). Furthermore, perturbation analyses artificially introducing a ± 2°C systematic temperature shift did not alter any major population-level conclusions, including inferred successful spawning hotspots and critical thermal windows (text S2.3). This robustness likely reflects the strong temporal constraint provided by hatch-date estimates and the fact that critical thermal windows were identified from modeled redd temperature histories rather than directly from individual otolith-based temperature reconstructions.

Although the subset of samples analyzed for otolith oxygen isotope measurements was necessarily smaller than the full sample set because of the labor-intensive nature of the analyses, supplementary analyses indicated that these individuals were broadly representative of the larger outmigrant population with respect to collection timing and hatch-date distributions (texts S1 and S7). Moreover, the primary ecological inferences in this study emerged from aggregated population-level spatial and temporal probability surfaces rather than from deterministic assignment of individual fish to specific redds. Nonetheless, because retrospective inference relies on sampled survivors, we cannot exclude the possibility that some juveniles originating from alternative thermal regimes were not represented in the analyzed subset. Accordingly, our conclusions should be interpreted as identifying the dominant patterns associated with observed survivors, rather than as an exhaustive reconstruction of all successful spawning environments.

Finally, implementation of the full framework presented here benefited from extensive environmental monitoring, redd surveys, temperature modeling, and isotopic characterization. As such, direct application of this exact workflow may be most feasible in river systems with existing population monitoring and well-resolved thermal and isotopic baselines. Application to other systems will require careful consideration of local isotope dynamics, thermal heterogeneity, and data availability, although the broader conceptual framework linking archival tissues, environmental reconstructions, and survival inference is adaptable to other temperature-sensitive species and river systems.

Future work that integrates retrospective reconstructions with prospective monitoring and mechanistic experiments will be particularly powerful, allowing managers to translate physiological thresholds into actionable flow and temperature strategies under a changing climate. Extending this framework across additional years with contrasting hydrologic and thermal regimes will help assess its robustness under variable conditions, while also clarifying the role of non-thermal stressors, such as discharge and dissolved oxygen, that may interact with temperature to influence early-life mortality. Likewise, applying it to adult survivors would provide a crucial test of whether the early-life constraints we identified in juveniles are reflected in the spawning population. More broadly, approaches that explicitly link developmental physiology, climate forcing, and management actions will be essential for anticipating and mitigating biodiversity loss in regulated freshwater ecosystems under continued warming.

## MATERIALS AND METHODS

### Fish collection

Winter-run juvenile Chinook salmon that successfully emerged and survived to the Red Bluff Diversion Dam (RBDD; Fig. 1) were provided by the United States Fish and Wildlife Service (USFWS). While rotary screw trap collections at RBDD occurred year-round in both 2021 and 2022, only fish that died incidentally during trapping were made available for this study. These incidental mortalities were collected between August and December of 2021 (*n* = 420) and 2022 (*n* = 125) (fig. S16), a period that corresponds to the peak passage timing of winter-run juveniles at RBDD (see text S7 for details). Patterns of incidental mortality collection closely mirror daily passage estimates at RBDD, indicating that these samples are broadly representative of the juvenile winter-run population in both years (text S7). Fork length and collection date data were available for all individuals. Fish were frozen at −20°C until otoliths were extracted. Collection and handling of fish were conducted under California Department of Fish and Wildlife (CDFW) CESA MOU (Lindley_12312), NOAA permit 17299-4R, and Institutional Animal Care and Use Committee (IACUC) protocol Johnr2309dn.

### Otolith microstructure analysis

Otolith microstructure analysis was performed to estimate hatch dates and the onset of exogenous feeding, which in winter-run Chinook salmon generally corresponds to fry emergence from the redd. Daily increments in the otolith were counted to estimate fish age and identify transitions between life stages. Specific landmarks corresponding to hatching and the onset of exogenous feeding (emergence) were used to back-calculate the number of days from capture to each life stage transition (fig. S17). A random subset of samples (*n* = 128 in 2021 and *n* = 96 in 2022) was selected for this analysis (see text S7).

Otolith thin sections were prepared following established protocols (*47*). Otoliths were first cleaned of adhering particles and rinsed with deionized ultrapure water, then stored dry until mounting. Each otolith was mounted on a glass round using Crystalbond 509 resin. The samples were ground on both sides along the sagittal plane using 600 and 1500 grit wet/dry sandpaper to expose the primordia and surrounding microstructure. The exposed surfaces were then polished using 3 μm and 1 μm Al_2_O_3_ lapping films. Finished samples were mounted to a coverslip using Gorilla Glue.

The dorsal side of each otolith was photographed at 20× magnification using a QImaging MicroPublisher 5.0 RTV digital camera mounted on an Olympus BX60 microscope, with ImagePro Premier software. Age estimation was performed independently by two trained readers. Daily increments were counted along a standardized 90° transect following Barnett-Johnson *et al.* (*47*). Agreement between readers was high with a mean absolute age difference of 3.2 days and a low coefficient of variation (CV) of 4.1%. Additional validation using known-age hatchery juveniles (*n* = 16) showed that otolith-derived age estimates closely matched expected ages with minimal systematic bias (mean signed error < 0.5 days; see text S8). These results support the assumption that otolith microincrements in juvenile Chinook salmon are deposited daily and that hatch-date estimates derived from otolith microstructure were reliable and reproducible. Age estimates from the first reader were used to back-calculate hatch and exogenous feeding dates.

### Water stable oxygen isotopes in the Sacramento River

To reconstruct early-stage temperature exposure in winter-run juvenile survivors, we measured the stable oxygen isotopic composition of water (δ^18^O_water_) at multiple locations in the Sacramento River (fig. S19). Water samples were collected directly from the river surface and stored in 20 mL glass top vials with Polyseal caps. Samples were collected during the 2021 and 2022 spawning seasons to match juvenile cohorts and spawning locations, and additional samples from 2024 were used to assess spatial and temporal variability. δ^18^O_water_ values were generally stable across sites and months (see text S9). To validate the use of surface water as a proxy for redd conditions in the gravel where embryos develop, we compared surface and subsurface δ^18^O_water_ in 2024 and found no significant differences (text S9), supporting the use of surface values to represent ambient conditions during early life stages.

Water samples were analyzed at the UC Davis Stable Isotope Facility using a Laser Water Isotope Analyzer V2 (Los Gatos Research, Inc., Mountain View, CA, USA). Each sample was analyzed at least eight times and the average of the last four injections was used to calculate the isotope ratio. During analysis, a sampling of 750 nL from a 0.5–1.5 mL sample was transferred to a heated injection block using a 2.0 μL syringe. Reference materials, including Vienna Standard Mean Ocean Water (VSMOW), Greenland Ice Sheet Precipitation (GISP), and Standard Light Antarctic Precipitation (SLAP), were used for standardization and calibration. The precision (1 standard deviation) of δ^18^O measurements for water samples was typically better than 0.1‰.

### Otolith stable oxygen isotope analysis

Stable oxygen isotope ratios (δ^18^O) in otoliths were measured using Secondary Ion Mass Spectrometry (SIMS) to reconstruct early-life temperature exposure. For isotope analysis, the second otolith from individuals used in microstructure analysis was randomly selected (*n* = 40 in 2021; *n* = 35 in 2022; text S7). Following the preparation method from Arai *et al.* (*25*), otoliths were embedded in West Systems 105 epoxy resin using individual cassette molds, sectioned with a low-speed diamond saw, and polished along the sagittal plane using 1500-grit sandpaper followed by 3, 1, and 0.1 μm aluminum oxide films. Polished otoliths were aggregated with calcite standards into multi-mount pucks, positioned to minimize geometric artifacts (*48*), then cleaned and gold-coated for SIMS analysis.

Oxygen isotope analyses were performed on the CAMECA ims-1290 SIMS at the University of California Los Angeles by using a focused Cs^+^ primary ion beam (∼2 nA) with 20 kV total accelerating voltage. To reduce the depth of the sputtered crater, a raster of 5 × 5 or 7 × 7 μm^2^ was applied during both presputtering (45 s) and data collection resulting in an analyzed area of approximately 15 μm in diameter. Dynamic transfer optics were applied and a normal incidence electron gun was used for charge compensation on the primary beam irradiated area. Negative secondary ions of ^16^O^−^ and ^18^O^−^ were detected simultaneously on Faraday cups (FC).

For SIMS δ^18^O measurements, we targeted the otolith region immediately preceding the exogenous feeding (emergence) check, corresponding to the pre-emergence alevin stage when juveniles are still occupying the natal redd (fig. S17). Multiple SIMS measurements (3–10 per otolith) were taken within this pre-emergence zone, which we identified as an optimal “sweet spot” for the most accurate early-life temperature reconstruction. This otolith region is (i) sufficiently distant from the otolith core to minimize maternal influences, organic content, and isotopic fractionation across the egg membrane (see text S5 for additional details), while (ii) still representing the redd-occupancy period prior to emergence, when ambient water δ^18^O can be spatially matched to natal redd conditions. In contrast, δ^18^O measurements from post-emergence zones are less reliable for accurate temperature reconstruction, as juveniles may have dispersed and experienced water δ^18^O outside their natal environment.

To correct for instrumental mass fractionation (IMF), we used UWC-3 calcite (*49*) (δ^18^O = 12.49‰ VSMOW) as the primary standard. UWC-3 was measured repeatedly throughout each analytical session and served both as the basis for IMF correction and as a running standard to monitor instrumental drift. When no drift was detected (generally defined as <0.25‰, 1SD, in the running standard across the session), the IMF correction was applied using the average isotope ratio of all UWC-3 analyses from that day. When drift was observed (>0.25‰, 1SD), the session was divided into brackets, and bracket-specific UWC-3 averages were applied for IMF correction. Although a few brackets (4 out of 30) slightly exceeded the 0.25‰ threshold, their inclusion is unlikely to affect overall results. Because the quantity of UWC-3 was limited, an additional calcite standard (UCLA_C2) was also measured in some sessions to monitor drift, but these were not used for IMF correction. The count rates of UWC-3 calcite were ∼3.0 × 10^9^ cps and ∼6.0 × 10^6^ cps for ^16^O and ^18^O, respectively. The reproducibility (2 standard deviation) of δ^18^O values of UWC-3 calcite was 0.3–0.6‰. The total error (${\mathrm{SE}}_{\text{total}}$) for each SIMS spot represents the analytical uncertainty as one standard error (SE) and was calculated as follows

$${\mathrm{SE}}_{\text{total}}=\sqrt{{\left({\mathrm{SEM}}_{\text{unknown}}\right)}^{2}+{\left({\mathrm{SEM}}_{\text{standard}}\right)}^{2}}$$ (1)

where ${\mathrm{SEM}}_{\text{unknown}}$ represents internal measurement precision (the standard error of the mean over six measured cycles), and ${\mathrm{SEM}}_{\text{standard}}$ represents external reproducibility (the SEM of bracketing calcite standards) from bracketing measurements.

After each analytical session, all SIMS spots were examined using images from the scanning electron microscope (Tescan Vega) at UCLA to confirm that they were not positioned on irregular surface features (e.g., cracks or breaks) that could bias the δ^18^O values. Any such spots were excluded from further consideration. Additionally, trends in ^16^O and ^18^O count rates over six analytical cycles were assessed for each spot to identify and exclude extreme outliers. All δ^18^O values were reported according to the international standard delta notation (permil, ‰). The delta notation reports the value relative to an international standard: VSMOW and Vienna Pee Dee Belemnite (VPDB) were used for water and carbonates, respectively.

### Temperature reconstruction from otolith stable oxygen isotopes

The temperature experienced by juveniles prior to emergence from the redd was reconstructed from otolith δ^18^O values measured by SIMS, combined with measured surface water δ^18^O from the Sacramento River, using the following Chinook salmon-specific fractionation equation calibrated for SIMS by Arai *et al.* (*25*)

$$\delta^{18}O_{\text{otolith}\left(\text{VPDB}\right)}-\delta^{18}O_{\text{water}\left(\text{VSMOW}\right)}=-0.14\times T({}^{\circ}C)+0.64$$ (2)

which can be rearranged to solve for temperature as

$$T\left({}^{\circ}C\right)=\frac{\delta^{18}O_{\text{water}\left(\text{VSMOW}\right)}-\delta^{18}O_{\text{otolith}\left(\text{VPDB}\right)}+0.64}{0.14}$$ (3)

To maximize the accuracy of temperature estimates, we matched both the spatial and temporal aspects of the water δ^18^O measurements to the juvenile cohorts. Specifically, we used δ^18^O measured in the primary spawning grounds of the Upper Sacramento River (stations WS5 and WS6; fig. S19) during the typical emergence period (summer to fall): surface water collected on September 25 for the 2021 samples and on October 15 for the 2022 samples. Notably, variation in water δ^18^O was relatively small across both space and time (see text S9 for details).

We validated otolith-based temperature reconstructions by comparing reconstructed temperatures from the otolith edge to measured water temperatures at the Red Bluff rotary screw trap where fish were collected. The comparison showed a positive relationship between otolith and environmental temperatures (slope = 1.24 ± 0.25 SE, *PP* < 0.001, adjusted R^2^ = 0.69), indicating overall agreement between environmental and otolith-based reconstructed temperature estimates (see text S6 for details).

### Aerial redd survey

To monitor the full potential spawning activity of winter-run Chinook salmon in 2021 and 2022, the spatial and temporal distribution of redds in the primary spawning habitat of the mainstem Sacramento River was determined from weekly aerial surveys conducted by the California Department of Fish and Wildlife (CDFW).

### River temperature model

The River Assessment for Forecasting Temperature (RAFT) model was used to estimate the water temperatures experienced by each redd observed during the aerial surveys. RAFT is a quantitative, process-based, one-dimensional river temperature model that simulates spatially explicit heat exchange processes along the longitudinal axis of the river, assuming laterally and vertically homogeneous temperatures within each river segment (*50*, *51*). The model internally simulates water temperatures at 10-min temporal resolution. For this study, modeled temperatures at each redd location were summarized as daily mean temperatures and daily standard deviations starting from the day each redd was first observed. These modeled temperature time series were then used to estimate hatch dates based on accumulated temperature units (ATU) and to quantify thermal exposure prior to emergence for each redd (see section below for details).

### Redd origin probability model

**Fig. 4.**
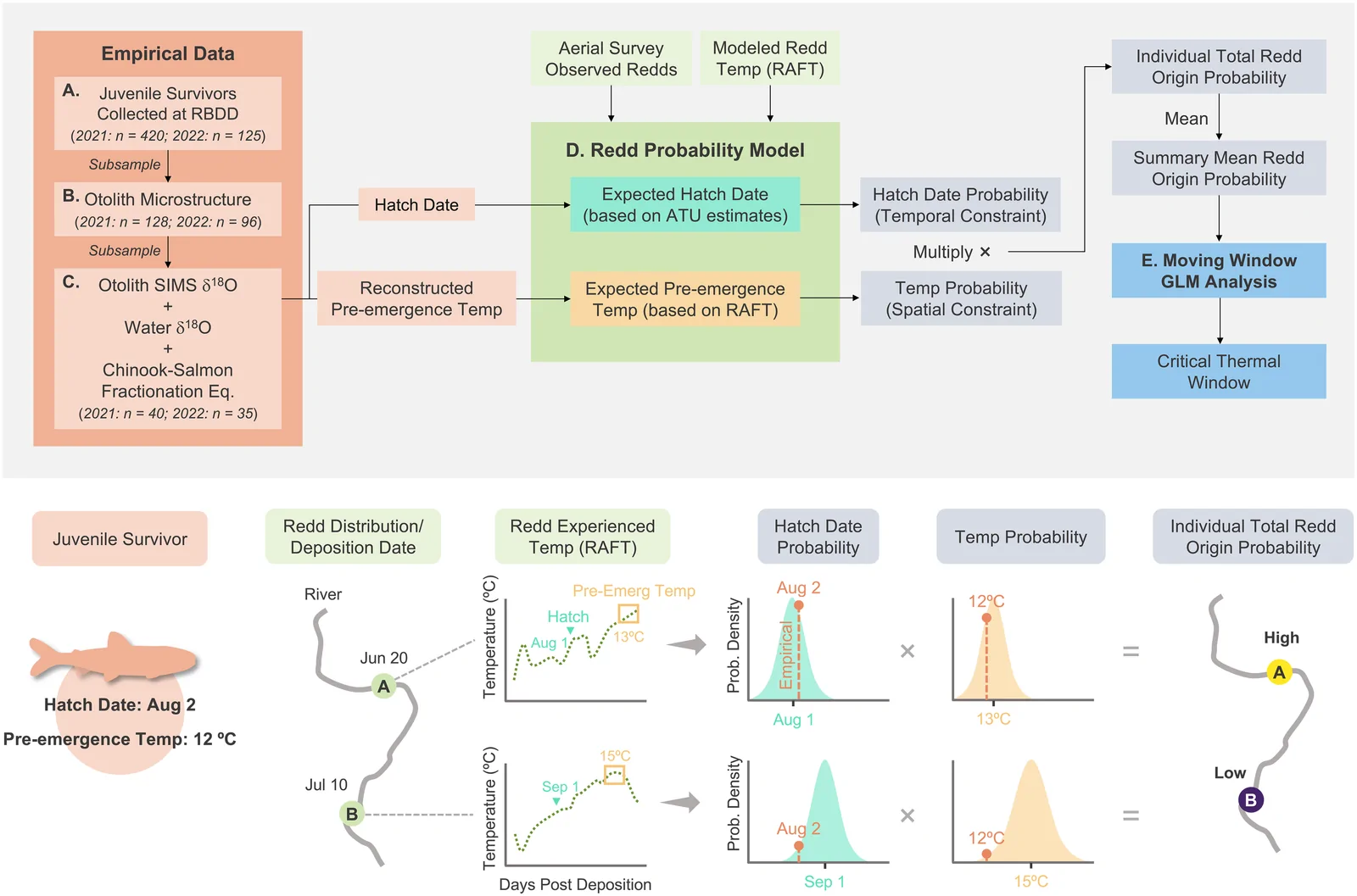
Analytical framework for reconstructing natal origins and identifying critical thermal windows associated with early-life survival. Top panel: Workflow summarizing the analytical framework used in this study and the sample sizes associated with each step. Juvenile winter-run Chinook salmon collected at RBDD (**A**) were subsampled for otolith microstructure analysis to estimate hatch dates (**B**) and paired otolith SIMS δ^18^O analysis to reconstruct pre-emergence temperatures (**C**). Final redd-origin probability analyses were conducted using the subset of individuals for which both hatch-date and otolith δ^18^O-derived temperature estimates were available (2021: *n* = 40; 2022: *n* = 35). These empirical data were integrated with aerial redd surveys and RAFT temperature modeling within the redd-origin probability framework (**D**) to estimate hatch-date and temperature-based probabilities of origin for each observed redd. Hatch-date estimates provided the temporal constraint on redd-origin assignment, whereas otolith-derived temperature estimates provided additional spatial discrimination among candidate redds. Hatch-date and temperature-based probability surfaces were then multiplied to generate individual total redd-origin probabilities, which were averaged across individuals to produce annual population-level heatmaps and subsequently used in a moving window GLM analysis (**E**) to identify critical thermal windows associated with recruitment success. Bottom panel: Example walkthrough of redd origin probability estimates for a survivor with an empirical otolith-derived hatch date of August 2 and a pre-emergence temperature of 12°C. For simplicity, probabilities are estimated for two redds [(A) and (B)] deposited on different dates. In this case, the redd probability model indicates a higher probability of origin from redd A. RBDD, Red Bluff Diversion Dam; SIMS, secondary ion mass spectrometry; RAFT, River Assessment for Forecasting Temperature model; ATU, accumulated temperature units; GLM, generalized linear model.

We developed a probability model to predict the geographic origin of redds for individual survivors based on empirical otolith-derived hatch dates and reconstructed pre-emergence temperatures (Fig. 4). Redd-origin probability analyses were conducted using the subset of juveniles for which both otolith microstructure-derived hatch dates and SIMS-based otolith δ^18^O temperature reconstructions were available (2021: *n* = 40; 2022: *n* = 35; total *N* = 75). For each individual, we calculated a probability density surface by combining two independent components: (i) a temporal constraint based on hatch dates (“hatch date probability”) and (ii) a spatial constraint based on pre-emergence temperatures (“temperature probability”). These two probability surfaces were multiplied to obtain a total spatiotemporal redd-origin probability surface for each individual.

To calculate the hatch-date based probability, we estimated, for each individual, the likelihood of originating from each observed redd given the correspondence between their otolith-predicted hatch date and the expected hatch date at that redd. Expected hatch dates were reconstructed using accumulated temperature units (ATU), assuming hatch occurred at 482 ATU following the winter-run Chinook salmon model (see text S10). Hatch dates at each redd were modeled as normally distributed, with the mean defined by the expected hatch date. Variance in hatch date incorporated three independent sources of uncertainty: (i) within-redd variance, reflecting biological variation in hatch timing among individuals within the same redd, assumed to be constant at 2.2 days across all redds (*52*); (ii) otolith ageing error, quantified as the coefficient of variation among two independent readers and allowed to vary among individuals; and (iii) uncertainty in the actual timing of egg deposition, arising from both the weekly interval of redd aerial surveys and behavioral lags between redd construction and egg laying. This deposition uncertainty was modeled as a bidirectional Gaussian error with a standard deviation of 2.8 days, derived by combining a 2-day SD from both survey timing and construction-to-deposition lag. These components were assumed independent and combined to yield the total hatch date uncertainty used in the model

$$\sigma_{\text{hatch},\text{redd}}=\sqrt{\sigma_{\text{within}-\text{redd}}^{2}+{\mathrm{CV}}_{\text{agei}ng\text{error}}^{2}+\sigma_{\text{deposition}}^{2}}$$ (4)

The posterior probability density of observing the individual’s otolith-based hatch date $x_{i}$ at ${\text{redd}}_{j}$ was then computed as

$$P_{\text{hatch},\mathrm{ij}}=\frac{1}{\sqrt{2\pi \sigma_{\text{hatch},\text{redd}}^{2}}}e^{\frac{-{\left(x_{i}-\mu_{\text{hatch},j}\right)}^{2}}{2\sigma_{\text{hatch},\text{redd}}^{2}}}$$ (5)

where $\mu_{\text{hatch},j}$ is the ATU-derived expected hatch date at ${\text{redd}}_{j}$.

The temperature-based probability was computed analogously, based on the assumption that an individual’s otolith-reconstructed pre-emergence temperatures reflect the temperatures experienced at its natal redd during the pre-emergence period. To estimate the relevant time window for each individual, we used otolith microstructure data and the SIMS spot locations along the dorsal and ventral axes. Specifically, we determined the minimum and maximum calendar dates corresponding to the shortest and longest SIMS spot distances from the otolith core, based on the position of the exogenous feeding check (representing the emergence date) and average daily increment widths (3.1 μm dorsal, 4 μm ventral axes). To account for the SIMS spot size (∼20 μm), we added three days to both the minimum and maximum dates. Across individuals, the reconstructed pre-emergence period had a median duration of 10 days (range: 7–32 days), with 81.3% of individuals having a reconstructed period of ≤15 days.

Using the estimated date range for each individual, we then calculated the expected mean pre-emergence temperature at each redd by averaging daily RAFT model temperature estimates over the individual-specific pre-emergence period. These mean expected temperatures at each redd were assumed to follow a normal distribution, with total variance incorporating both fixed (global) and individual-specific sources of uncertainty. Fixed uncertainty terms applied equally across individuals and included: (i) otolith temperature prediction uncertainty associated with the temperature-fractionation equation [cross-validated RMSE = 1.97°C (*25*)]; (ii) seasonal variation in Sacramento River water δ^18^O values, estimated using all available measurements from the primary spawning region (WS5–WS6) across spawning season (July–October) and years (2021, 2022, and 2024) and converted to temperature scale (SD = 0.91°C); and (iii) RAFT model prediction error (RMSE = 0.2°C). Individual-specific uncertainty terms included: (iv) SIMS analytical uncertainty, including within-sample internal measurement precision and external reproducibility; (v) daily uncertainty in RAFT temperature estimates; and (vi) temporal variability in mean RAFT-derived temperatures across each individual’s inferred pre-emergence period. Assuming independence among uncertainty components, these sources were combined to estimate the total uncertainty of the temperature model

$$\begin{array}{c} \sigma_{\text{temp},\text{redd}}\\ =\sqrt{{\text{RMSE}}_{\text{otolith prediction}}^{2}+\sigma_{\text{water}\delta^{18}O}^{2}+{\text{RMSE}}_{\text{RAFT}}^{2}+\sigma_{\text{SIMS}}^{2}+\sigma_{\text{RAFT daily}}^{2}+\sigma_{\text{RAFT period}}^{2}} \end{array}$$ (6)

The posterior probability density of observing the individual’s otolith-based mean pre-emergence temperature $y_{i}$ at ${\text{redd}}_{j}$ was then computed as

$$P_{\text{temp},\mathrm{ij}}=\frac{1}{\sqrt{2\pi \sigma_{\text{temp},\text{redd}}^{2}}}e^{\frac{-{\left(y_{i}-\mu_{\text{temp},j}\right)}^{2}}{2\sigma_{\text{temp},\text{redd}}^{2}}}$$ (7)

where $\mu_{\text{temp},j}$ is the mean RAFT-predicted temperature for the pre-emergence period at ${\text{redd}}_{j}$.

To estimate the natal origin of each juvenile, we combined the independent probability surfaces from hatch dates and temperature. For each individual i and candidate ${\text{redd}}_{j}$, we first calculated the joint probability of origin as the product of the hatch date and temperature probabilities under the assumption that these two components are independent and contribute equally

$$P_{\mathrm{ij}}^{*}=P_{\text{hatch},\mathrm{ij}}\times P_{\text{temp},\mathrm{ij}}$$ (8)

These unnormalized joint probabilities were then normalized across all candidate redds for each individual such that the final total probability surface summed to one

$$P_{\text{total},\mathrm{ij}}=\frac{P_{\mathrm{ij}}^{*}}{\sum_{k}P_{ik}^{*}}$$ (9)

This yielded a redd-level probability surface for each individual, where $P_{\text{total},\mathrm{ij}}$ represents the probability that juvenile i originated from ${\text{redd}}_{j}$.

To identify population-level “hotspots” of successful spawning (i.e., redds that produced juveniles that survived and were captured downstream), we computed the *mean redd origin probability* for each redd in each year

$$\bar{p_{j}}=\frac{1}{N}\sum_{i=1}^{N}P_{\text{total},\mathrm{ij}}$$ (10)

where N is the number of juveniles sampled in that year. This produced annual summary heatmaps, where each redd reflects its average probability of being the natal origin of juveniles that successfully emerged and survived to the downstream collection site.

We validated the redd probability model using a unique subset of five juvenile survivors collected at RBDD for which hatch date estimates, otolith δ^18^O-based temperature reconstructions, and parentage-based tag (PBT) matches with adult spawners were all available. The PBT matches provided independent estimates of the spawning location and timing of each juvenile’s parent, allowing direct comparison with redd origin estimates derived from the otolith-informed redd probability model. This comparison served as an external validation of model performance (see text S4 for details).

### Moving window GLMs

To identify the life stage most sensitive to temperature-dependent mortality (i.e., the “critical thermal window”), we related mean redd origin probabilities from the redd probability model to daily redd temperature time series from the RAFT model. This analysis allowed us to objectively determine (i) the developmental period when survival is most affected by high temperatures and (ii) the approximate temperature at which mortality risk increases.

We implemented a moving window generalized linear model (GLM) analysis, systematically varying the start and end of the time window post redd deposition to examine temperature effects across early life stages. We conducted the analysis on aggregated redd groups, where redds sharing the same observation date and spatial location were combined. Modeling at the individual redd level in cases where redds have identical values for both response and predictor variables treats these points as independent observations, which underestimates standard errors and increases the risk of overconfident inference and Type I errors. Specifically, we tested windows starting at 1, 8, 15, … up to 71 days post-deposition and ending at 7, 14, 21, … up to 84 days (total of 77 combinations). We set the minimum window interval to be 7 days, which matches the temporal resolution of redd survey observations and minimizes bias associated with uncertain redd deposition timing (SD = 2.8 days).

For each start-end combination, we calculated the mean temperature experienced by each redd group within the window. We then fit a GLM predicting the mean redd origin probability (i.e., inferred survival) as a function of mean temperature and included redd number (i.e., the counts of redds in each group) as an additional covariate to account for the influence of redd abundance on the mean redd origin probability. Year was also included as a categorical predictor to control for interannual differences between 2021 and 2022. A gamma distribution with a log link function was used to appropriately model the positive continuous probability response.

Model fit was assessed using Akaike’s Information Criterion (AIC), and ΔAIC values relative to the best-fitting model were visualized as a heatmap. The window with the lowest AIC identifies the developmental period most strongly associated with temperature-dependent mortality.

We evaluated the robustness of both the population-level mortality hotspots and moving window GLM analyses to sampling variation using bootstrap resampling procedures (texts S2.1 and S2.2). We additionally performed perturbation analyses to test the robustness of inferred spawning hotspots and critical thermal windows to large systematic shifts in predicted temperatures and hatch dates (text S2.3). Furthermore, to evaluate whether temperature sensitivity aligned more closely with developmental stage, we repeated the moving window analysis using accumulated temperature units (ATU) instead of days post-deposition (text S3). All analyses were carried out in R version 4.6.0.

## Supplementary Materials

### This PDF file includes:

Supplementary Text S1 to S10 Figs. S1 to S20 References
