# Supplementary material for: Thermal bottlenecks constrain early-life survival of native fish in regulated rivers
Source: Sci Adv. 2026 Aug 21;12(34):eaeg3586. doi: 10.1126/sciadv.aeg3586 (PMC13496188; doi:10.1126/sciadv.aeg3586)
Supplement: Supplementary file 1 [file sciadv.aeg3586_sm.pdf]

Science Advances
AAAS

Supplementary Materials for

Thermal bottlenecks constrain early-life survival of native fish in regulated rivers

Kohma Arai *et al.*

Corresponding author: Kohma Arai, kohmaarai@gmail.com

Sci. Adv. **12**, eaeg3586 (2026)
DOI: 10.1126/sciadv.aeg3586

This PDF file includes:

Supplementary Text S1 to S10
Figs. S1 to S20
References

Supplementary Text

Text S1. Otolith-based hatch dates and early-stage temperature reconstruction

The ages of juvenile winter-run Chinook salmon collected at Red Bluff Diversion Dam were estimated based on total daily otolith increment counts from the hatch check to the otolith edge at collection. Median estimated age was 49 days for the 2021 otolith microstructure subsample (n = 128) and 56 days for the subset analyzed for $\delta^{18}O$ (n = 40), while corresponding median ages in 2022 were 50 days (n = 96) and 55 days (n = 35), respectively.

Hatch dates were then back-calculated from daily otolith growth increments. For 2021 (warm year), the median hatch date was 7 August (n = 128; Fig. S2), and for the subset measured for $\delta^{18}O$ (n = 40) the median was 3 August, consistent with the full subset. In 2022 (cool year), the median hatch date was 17 August (n = 96), with a median of 19 August for the $\delta^{18}O$ subset (n = 35). Surviving juveniles from 2021 hatched approximately 10 days earlier than those from 2022 ($p < 0.001$; Fig. S2). Hatch timing in 2021 peaked sharply in early August, whereas 2022 exhibited a broader, later-spanning distribution.

Otolith stable oxygen isotope ($\delta^{18}O$) values measured using secondary ion mass spectrometry (SIMS) varied among individuals and between years (Fig. S3A). These otolith $\delta^{18}O$ measurements were then converted to water temperatures experienced prior to redd emergence using water $\delta^{18}O$ estimates and established fractionation relationships (*25*) (Fig. S3B). Reconstructed temperatures also varied among individuals, with survivors in 2021 experiencing higher mean pre-emergence temperatures (11.44 ± 0.56 °C) than those in 2022 (10.44 ± 0.59 °C).

Text S2. Sensitivity analyses

Text S2.1. Population-level summary heatmap bootstrap analysis

To evaluate the robustness of estimated mean redd origin probabilities ("mortality hotspots") to sampling variation, we conducted a non-parametric bootstrap analysis. For each bootstrap replicate, we resampled fish with replacement and calculated the mean natal origin probability across sampled fish. We repeated this procedure 1,000 times to generate bootstrap distributions of the mean and standard deviation.

Bootstrap results demonstrated that in both 2021 and 2022, redd groups with higher mean origin probabilities tended to exhibit lower bootstrap standard deviations (Fig. S6). This indicates that the strongest signals of natal origin were also the most stable and robust to resampling of individuals. In contrast, redd groups with low or intermediate mean origin probabilities showed comparatively greater variability across bootstrap replicates, reflecting higher sensitivity to the individuals included in the analysis. Overall, these results suggest that the most prominent natal origin signals in the dataset are unlikely to be artifacts of sampling variation.

Text S2.2. Moving window GLM bootstrap analysis

To assess the robustness of the optimal thermal window to sample composition, we performed a bootstrap analysis in which 20% of redd groups were randomly held out in each iteration. The moving window generalized linear model (GLM) analysis was then repeated on the remaining data. This procedure was performed 100 times to evaluate the consistency of window selection across bootstrap replicates.

Results from the bootstrap analysis closely matched those obtained with the full dataset. The 1–14 day post-deposition window was consistently identified as the top-performing model, followed closely by the 1–7 day window (Fig. S7). Windows spanning 1–21 and 1–28 days also performed well, though with slightly higher Akaike Information Criterion (AIC) values. Beyond these early-life windows, model

performance plateaued and all other window combinations showed substantially higher AICs. Together, these results demonstrate that critical early developmental periods (particularly within the first two weeks post-deposition) were consistently identified as top-performing models across bootstrap replicates, indicating strong robustness to redd-level sampling variability.

Text S2.3. Perturbation analysis

To evaluate the sensitivity of inferred redd-origin distributions (Fig. 2) and critical thermal window (Fig. 3) analyses to uncertainty in reconstructed temperatures and hatch-date estimation, we conducted a perturbation analysis that systematically altered reconstructed temperatures and hatch dates for all individuals prior to model fitting. Specifically, reconstructed temperatures derived from otolith $\delta^{18}O$ were shifted by ±2 °C and hatch dates estimated from otolith microstructure analysis were shifted by ±3 d relative to the baseline dataset. These perturbations were selected as conservative stress-test scenarios that substantially exceed empirically observed uncertainty in the underlying ageing and temperature reconstructions.

For each perturbation scenario, we re-ran the redd-origin probability model and recalculated population-level summaries of inferred redd origin distributions. We then quantified similarity between perturbation and baseline scenarios using Pearson correlations of redd-origin probability surfaces across space and time. We additionally repeated the moving window generalized linear model (GLM) analysis used to identify the developmental period most sensitive to thermal stress.

Perturbation analyses demonstrated that the major spatial and temporal patterns of inferred successful spawning locations were highly robust to systematic perturbations in reconstructed temperature and hatch timing (Fig. S8). Temperature perturbation scenarios (±2 °C) produced nearly identical redd-origin distributions relative to the baseline scenario (r = 0.984–0.995; Fig. S8), indicating that even large systematic shifts in reconstructed temperature minimally altered the inferred successful reproduction

hotspots. Hatch-date perturbations produced moderately strong correlations with the baseline scenario (*r* = 0.674–0.814; Fig. S8), indicating some sensitivity of inferred redd-origin distributions to systematic timing displacement, although the dominant spatial and temporal hotspot patterns remained broadly consistent across scenarios.

Similarly, inferred critical developmental windows were highly robust to temperature perturbations. Under both ±2 °C scenarios, the best-supported moving window remained days 1–14 post deposition, consistent with the baseline analysis (Fig. S9). The −3 d hatch-date perturbation modestly shifted the optimal developmental window to days 1–28 post deposition, which remained largely consistent with the baseline inference. In contrast, the +3 d hatch-date perturbation identified a substantially later developmental window (days 71–84 post deposition) as the nominal best-supported model. However, these later-window models were associated with positive temperature coefficients, opposite in sign to the negative relationship between temperature and survival observed in the baseline analysis. This reversal suggests that the later-window support likely reflects temporal misalignment artifacts introduced by imposing unrealistic systematic hatch-date displacement rather than evidence for biologically meaningful late-stage thermal sensitivity.

Overall, these analyses indicate that the primary conclusions of this study are highly robust to uncertainty in reconstructed temperatures and reasonably robust to hatch-date uncertainty. Importantly, independent validation using known-age hatchery fish demonstrated that otolith increment counts exhibited minimal systematic directional bias across readers (mean signed error < 0.5 d; see Text S8), substantially smaller than the ±3 d perturbation scenarios evaluated here. Furthermore, uncertainty associated with hatch-date estimation and temperature reconstruction was explicitly propagated through the probabilistic redd-origin assignment framework (see main text in Materials and Methods section). Together, these results suggest that the perturbation scenarios evaluated here represent conservative stress tests rather than realistic

sources of systematic error, and that the primary ecological inferences of this study remain well supported.

Text S3. Moving window GLM using accumulated temperature units (ATU)

To evaluate whether thermal sensitivity aligned more closely with developmental stage rather than calendar time, we repeated the moving window generalized linear model (GLM) analysis using accumulated temperature units (ATU) instead of days post-deposition. ATU provides a temperature-dependent measure of developmental progress, which is more biologically meaningful for incubation processes whose rates are largely governed by thermal accumulation rather than elapsed days (*53*).

The analytical procedure followed the same framework described in the main Materials and Methods, with modifications for ATU. Briefly, daily temperatures from the RAFT model were converted to cumulative ATU for each redd group. We then evaluated windows starting at 0, 70, 140, … up to 770 ATU and ending at 70, 140, 210, … up to 840 ATU. For each window, we calculated the mean temperature experienced by each redd group and fit a GLM predicting mean redd-origin probability as a function of mean temperature, with redd count included as a covariate to account for group size effects. Year (2021 vs. 2022) was included as a categorical predictor to account for interannual differences. A gamma GLM with a log link was used to model the positive continuous probability response.

Model support was assessed using Akaike's Information Criterion (AIC), and ΔAIC values relative to the best model were visualized as a heatmap. To evaluate robustness to sample composition, we conducted a bootstrap analysis in which 20% of redd groups were removed at random and the moving window analysis was repeated. This procedure was repeated 100 times to assess the stability of window selection.

Results from the ATU-based analysis closely matched those from the calendar-day approach. Across most bootstrap iterations, the 0–140 ATU window had the lowest AIC, followed closely by the 0–70 and 0–210 ATU windows (Fig. S10). These windows correspond to the very early embryonic period, including fertilization through gastrulation. Together, these results suggest that the highest temperature sensitivity

could be occurring within a narrow early-life window and that this finding is robust whether developmental time is indexed using days post-deposition or thermal accumulation.

Text S4. Redd probability model validation based on parentage-based tags (PBT)

To validate the accuracy of the otolith-informed redd origin probability model, we developed an independent spatiotemporal probability model using parentage-based tag (PBT) assignments linking juvenile survivors to known spawning adults. We used the probability density surface resulting from this PBT-informed model to compare with that of the otolith-informed model and assessed agreement between approaches. Adult winter-run Chinook salmon carcasses in 2021 and 2022 were collected by the United States Fish and Wildlife Service (USFWS), Pacific States Marine Fisheries Commission (PSMFC), and California Department of Fish and Wildlife (CDFW) from May to September. For each adult carcass encountered, a fin clip was obtained and the sex, carcass freshness, recovery date, and carcass recovery location was recorded. Juveniles collected at the downstream rotary screw trap also had fin clips collected. Tissues from adult carcasses and juveniles were sent to Cramer Fish Sciences for genotyping. Juvenile genotypes were compared to adult carcass genotypes using kinship analysis to identify parental matches (see Blankenship et al. (*54*) for detailed methods). Five juveniles included in the oxygen isotope dataset (n = 75) were successfully matched to at least one parent (2021 n =4; 2022 n =1). These matches represented four effective spawning adults because one male was linked to two juveniles.

For each PBT-linked adult, we constructed a spatiotemporal redd-origin probability surface by combining two independent components: (i) a temporal probability surface based on carcass recovery date ("carcass date probability") and (ii) a spatial probability surface based on carcass recovery location ("carcass location probability"). These two probability surfaces were multiplied and normalized across all candidate redds to generate a final relative probability surface describing the most likely redd origin associated with each adult–juvenile pair.

To estimate the carcass-date based probability, we calculated, for each redd, the likelihood of observing the carcass recovery date given the expected interval between spawning and carcass recovery. Expected

carcass recovery dates were estimated from aerial redd survey deposition dates plus mean carcass residence time derived from published observations of female spring-run Chinook salmon (*55*), assuming a normal distribution (mean = 12 days; SD = 5 days). Although males do not guard redds, we assumed freshly recovered male carcasses could have originated from redds within the same approximate temporal window as well. Variance in carcass-date probability incorporated three independent sources of uncertainty: (i) carcass residence-time variance, reflecting biological variability in the interval between redd construction and carcass recovery (SD = 5 days); (ii) aerial redd survey timing uncertainty, arising from the weekly interval between redd surveys (SD = 2 days); and (iii) uncertainty in the interval between redd construction and actual egg deposition (SD = 2 days). Following the hatch-date probability framework described in the main text (see "*Redd origin probability model*" in the Materials and Methods), survey timing and construction-to-deposition uncertainty were combined to estimate a bidirectional redd deposition uncertainty with an SD of 2.8 days. These uncertainty components were assumed independent and combined to yield the total carcass-date variance used in the model.

To estimate the carcass-location based probability, we calculated the likelihood of each redd given the correspondence between carcass recovery river kilometer and the expected carcass recovery location associated with that redd after accounting for downstream carcass drift. Expected carcass locations were estimated using sex-specific downstream drift distances derived from winter-run Chinook salmon carcass mark–recapture studies (*56*) assuming normal distributions. Female carcasses were assigned a mean downstream drift distance of 1.069 km (SD = 1.22 km), whereas male carcasses were assigned a mean drift distance of 2.2 km (SD = 3.52 km). The larger variance assigned to males reflected lower spawning-site fidelity and greater uncertainty in movement prior to death.

Posterior probability densities in both time and space were then calculated using Gaussian probability density functions following the same general framework described in the main text. Probabilities were constrained to biologically plausible redds only (e.g., redds occurring after carcass recovery or

downstream of carcass location were assigned zero probability). The resulting temporal and spatial probability surfaces were multiplied to generate the final spatiotemporal redd-origin probability surface for each PBT-linked fish.

To quantify agreement between the otolith-informed and PBT-informed models, we applied a series of percentile-based thresholds (ranging from 0.01 to 0.5) to classify redds as either "likely" or "unlikely" origins. Redds with probabilities within the upper percentile (e.g., top 20%) were designated as "likely" candidate origins. A "match" was defined as any case in which at least one redd identified as "likely" by the otolith-informed model overlapped with a redd identified as "likely" by the PBT-informed model for a given fish. The proportion of fish with at least one such match provided a measure of agreement, while the percentile threshold itself represented precision, as it determined how many redds were labeled as "likely." Because there is typically a trade-off between agreement and precision—stringent thresholds (high precision) yield fewer likely redds and can reduce agreement—we assessed model agreement across the full range of thresholds to identify the best balance between the two metrics (Fig. S11).

The upper 20% threshold produced the most balanced trade-off, yielding overlapping redd assignments between models for four of five fish (80% agreement). To visualize agreement at this threshold, we mapped redds in space and time, highlighting those identified as overlapping between models, selected only by the PBT-informed model, selected only by the otolith-informed model, or unselected by either model (Fig. S12). Overall, the two independent approaches showed generally consistent spatiotemporal patterns, although the degree of overlap varied among individuals. Some fish exhibited close correspondence in inferred spawning timing and location between models, whereas others showed broader or only partial overlap. Accordingly, the otolith-informed reconstructions should be interpreted as probabilistic estimates that were broadly consistent with, but not identical to, the independent PBT-informed assignments.

Finally, to assess whether the observed agreement between the otolith-informed and PBT-informed models exceeded what would be expected by random chance, we constructed a null distribution of the total number of redd overlaps across all fish for each probability threshold. For each iteration, we randomly reshuffled the sets of top-ranked ("likely") redds for both models while preserving the observed number of candidate redds per fish, and then calculated the total number of overlapping redds. This process was repeated 100,000 times to generate the null expectation. The observed overlap between models consistently exceeded the random expectation across all thresholds examined (0.05–0.2; Fig. S13), indicating that the agreement between the two models was unlikely to have occurred by chance.

Text S5. Early-stage temperature reconstruction validation using known temperature tank fish

Oxygen isotope values near the otolith core can be influenced by maternal inputs, early-life physiology, and other non-environmental factors (*57*). This is particularly problematic in species with large eggs or when maternal and offspring environments differ markedly at the start of life (e.g., anadromous Chinook salmon). However, the exact developmental point—i.e., the distance from the core—at which these maternal and physiological influences diminish and otolith-based temperature reconstructions begin to reliably reflect environmental conditions remains poorly understood.

To identify when in early development otolith-based temperature reconstructions become reliable, we analyzed otoliths from tank-reared juvenile Chinook salmon (n = 6) held at a constant, known temperature (~10 °C) with measured water $\delta^{18}O$. These fish, acquired as eyed embryos from the Nimbus Fish Hatchery (American River, California, USA), were reared under controlled conditions from the eyed-egg stage through the onset of exogenous feeding. Otolith $\delta^{18}O$ values were measured using secondary ion mass spectrometry (SIMS) along a continuous transect from the core (primordium) to the edge. Using the known water $\delta^{18}O$ and a Chinook-specific temperature–dependent fractionation equation (*25*), we reconstructed ambient temperatures along the otolith transect.

We calculated one-sample z-scores at each measurement point to test whether reconstructed temperatures differed significantly from the known tank temperature. Z-scores were computed using the predicted temperature and its associated standard error. Results showed that temperature reconstructions within ~100 µm of the core often substantially overestimated the known temperature, with z-scores exceeding the significance threshold ($|z| > 1.96$; Fig. S14). In contrast, measurements beyond ~120 µm generally aligned with the known temperature, producing z-scores within the non-significant range and indicating accurate, reliable estimates. These findings suggest that otolith-based temperature reconstructions of

Chinook salmon become generally reliable at distances greater than approximately 120 μm from the otolith core along the dorsal edge.

Text S6. Otolith-based temperature reconstruction validation using otolith edge measurements

To validate temperature reconstructions from otolith stable oxygen isotopes ($\delta^{18}O$), we compared reconstructed temperatures at or near the otolith edge to known water temperatures at the time of fish collection at Red Bluff Diversion Dam (RBDD) rotary screw trap. Daily water temperatures at Red Bluff were averaged over a 7-day window ending on the fish collection date to provide a smoothed environmental reference. For each otolith, we calculated weighted mean temperature for the region closest to the edge (i.e., post-exogenous or edge region), where individual secondary ion mass spectrometry (SIMS) spots were weighted by the inverse of their offset (in days) from the collection date—approximated using distance of the spot from the otolith edge—to emphasize values closest to the time of capture. Otoliths whose nearest edge spots were estimated to be more than 7 days from the collection date were excluded, resulting in a final sample size of $n = 12$. Despite the small sample size, this dataset encompassed a broad range of water temperatures at Red Bluff (9.1–16.4 °C), providing a suitable basis for comparison with otolith-based reconstructions.

We expected a 1:1 relationship between otolith-derived temperatures and Red Bluff temperature if reconstructions accurately reflected recent thermal exposure. To assess this, we fit a linear model relating weighted otolith temperature to 7-day Red Bluff average temperature (Fig. S15). The resulting model showed a positive relationship (slope = 1.24 ± 0.25 SE, $p < 0.001$), with moderate explanatory power (adjusted $R^2 = 0.69$). We expected some individual-level deviation from a 1:1 relationship because otolith-derived temperatures integrate thermal exposure over the period represented by the sampled otolith material, whereas environmental temperatures were based on river conditions at Red Bluff near the time of capture. During this interval, juveniles may have moved substantial distances and experienced a range of temperatures not fully captured by the local environmental record. Despite this individual-level variability, a *t*-test indicated that the estimated slope did not differ significantly from 1 ($t = 0.98$, $p = 0.35$), and the intercept did not differ significantly from 0 ($t = –0.97$, $p = 0.36$), supporting the absence of

systematic bias. Together, these results indicate overall agreement between environmental and otolith-based reconstructed temperature estimates.

Text S7. Assessment of sample representativeness

To evaluate whether the winter-run juvenile Chinook salmon samples collected as incidental mortalities at the Red Bluff Diversion Dam in 2021 and 2022, as well as the subsets analyzed for otolith microstructure and stable oxygen isotopes were representative of the broader outmigrating population, we compared our sample distributions to daily passage estimates from the SacPAS database (www.cbr.washington.edu/sacramento/). Specifically, we assessed the distributions of collection date and fork length across the full incidental mortality dataset, the otolith microstructure subset, and the stable oxygen isotope subset. For each group, we compared the median, interquartile range (IQR), and 95% range, and used Kolmogorov–Smirnov (KS) tests to determine whether the distributions differed statistically.

In both 2021 and 2022, the temporal pattern of sample collection closely matched the daily passage estimates at Red Bluff (Fig. S16A). In 2021, peak passage occurred in late September to early October, with a secondary peak in early November. In 2022, passage peaked in late September and early November. The distribution of collection dates for the full sample of incidental mortalities mirrored this seasonal trend, and both the otolith microstructure and stable isotope subsamples aligned well with these temporal patterns. Median collection dates were consistent across all groups and KS tests confirmed no statistically significant differences in collection date distributions between the full dataset and either subsample in both years ($p > 0.05$).

Fork length distributions showed some divergence in 2021, where the full sample had a smaller median fork length compared to the otolith microstructure and isotope subsamples (Fig. S16B; $p < 0.01$). However, in 2022, fork length distributions were statistically similar across all groups ($p > 0.05$). Despite the slight size difference observed in 2021, the temporal agreement and largely consistent distributions

support the conclusion that our analyzed samples are broadly representative of the full juvenile winter-run Chinook salmon population passing Red Bluff in both years.

Text S8. Age validation using known-age hatchery fish

To validate otolith-based age estimation, we analyzed otoliths from juvenile Chinook salmon of known age obtained from Coleman National Fish Hatchery (*n* = 16). Because exact hatch dates were unavailable for individual fish, "true age" was defined as the number of days between ponding and collection. Ponding closely corresponded to the onset of exogenous feeding and produced a distinct otolith check that was consistently identifiable by readers. Previous observations of fry sampled shortly after ponding further supported that daily otolith increments were deposited consistently following this check.

Three independent readers each conducted two independent otolith reads on the known-age samples. Estimated ages closely matched expected ages across readers, with minimal systematic bias (mean signed error < 0.5 d; Fig. S18A). Error distributions were centered near zero, indicating no evidence of consistent over- or under-ageing bias (Fig S18B).

These results support the assumption that otolith microincrements in juvenile Chinook salmon are deposited daily and indicate that hatch-date estimates derived from otolith microstructure were accurate.

Text S9. Sacramento River water stable oxygen isotopes

To assess spatiotemporal trends in water stable oxygen isotope values ($\delta^{18}O$) within the Upper Sacramento River watershed, we collected surface water samples from nine sites, including six mainstem stations and three tributary sites (Fig. S19A). For early-life temperature reconstructions of juveniles collected in 2021 and 2022, we used $\delta^{18}O$ values collected from mainstem stations WS5 and WS6, located near the primary spawning habitat, during the fall emergence period to best approximate the water conditions experienced by fish.

While tributary sites (WS3, WS8, and WS9) exhibited some variation, mainstem $\delta^{18}O$ values were highly stable (Fig. S19B). Across all sampling years, mean $\delta^{18}O$ ranged from –11.80 to –11.92‰ among stations, with within-site standard deviations ≤0.27‰, indicating low spatiotemporal variability.

To evaluate whether surface water $\delta^{18}O$ is a suitable proxy for the conditions experienced by developing fish within redds, we compared paired surface and subsurface water samples collected from the Upper Sacramento River between July–October and December 2024 (Fig. S20). A two-way ANOVA with water type (surface vs. subsurface), collection month, and their interaction as factors revealed no significant effect of water type ($p = 0.74$) or its interaction with month ($p = 0.96$), but a significant effect of collection month ($p < 0.001$). Thus, seasonal shifts were detected, but surface and subsurface $\delta^{18}O$ values did not differ systematically. These findings indicate that surface water $\delta^{18}O$ provides a reliable proxy for subsurface conditions during early life stages, and the overall stability of mainstem $\delta^{18}O$ makes bias in otolith-based temperature reconstructions unlikely.

Text S10. Winter-run specific accumulated temperature units for hatch date estimation

We calculated the median accumulated temperature units (ATU) to hatch for winter-run Chinook salmon using daily water temperatures and hatch observations from streamside incubation of winter-run Chinook in the McCloud River in 2023 and 2024. Eggs were taken at the eyed stage from Livingston Stone National Fish Hatchery (LSNFH), after approximately 302 ATU and delivered to streamside heath trays. For four heath trays across the two years, the earliest and latest date of hatching was reported, giving us a minimum, maximum, and median date of hatch for each heath tray (the number of eggs hatched was not recorded). We summed daily water temperatures fish experienced to hatch for early, median, late dates and added this to the 302 ATU already experienced at the hatchery. Across the four time series, the mean of the median ATU was 486 ATU, which aligned with LSNFH estimates of ATU to hatch (482 ATU). We ultimately used the 482 ATU in our model for estimating hatch dates of each redd, given that this value has been refined through years of hatchery rearing, but provide these lines of evidence from fish reared streamside to support the use of this value over the wider range found in the literature (400–526 ATU).

Supplementary Figures

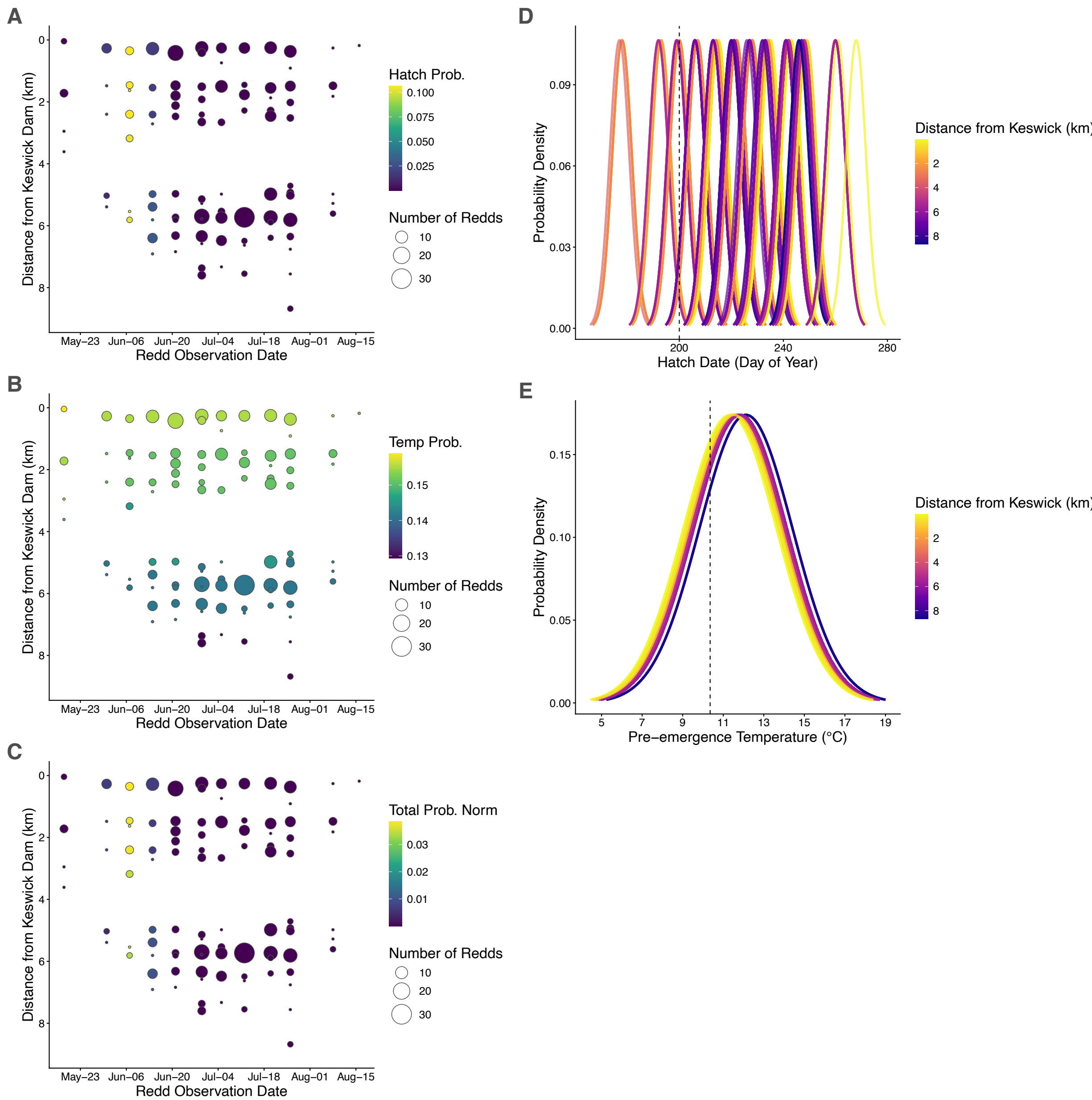

Fig. S1 | Example redd-origin probability model output. Example model outputs for an individual juvenile Chinook salmon collected at the Red Bluff Diversion Dam on 2022-09-21, with a reconstructed hatch date of 2022-07-19 and pre-emergence otolith-based temperature of 10.4°C. Panels show: (**A**) hatch date probabilities, (**B**) temperature probabilities, and (**C**) normalized total probabilities. Also shown are (**D**) the expected hatch date distribution across all potential redds, and (**E**) the expected pre-emergence temperature distribution across those redds, with vertical dashed lines indicating observed empirical hatch date and pre-emergence temperature.

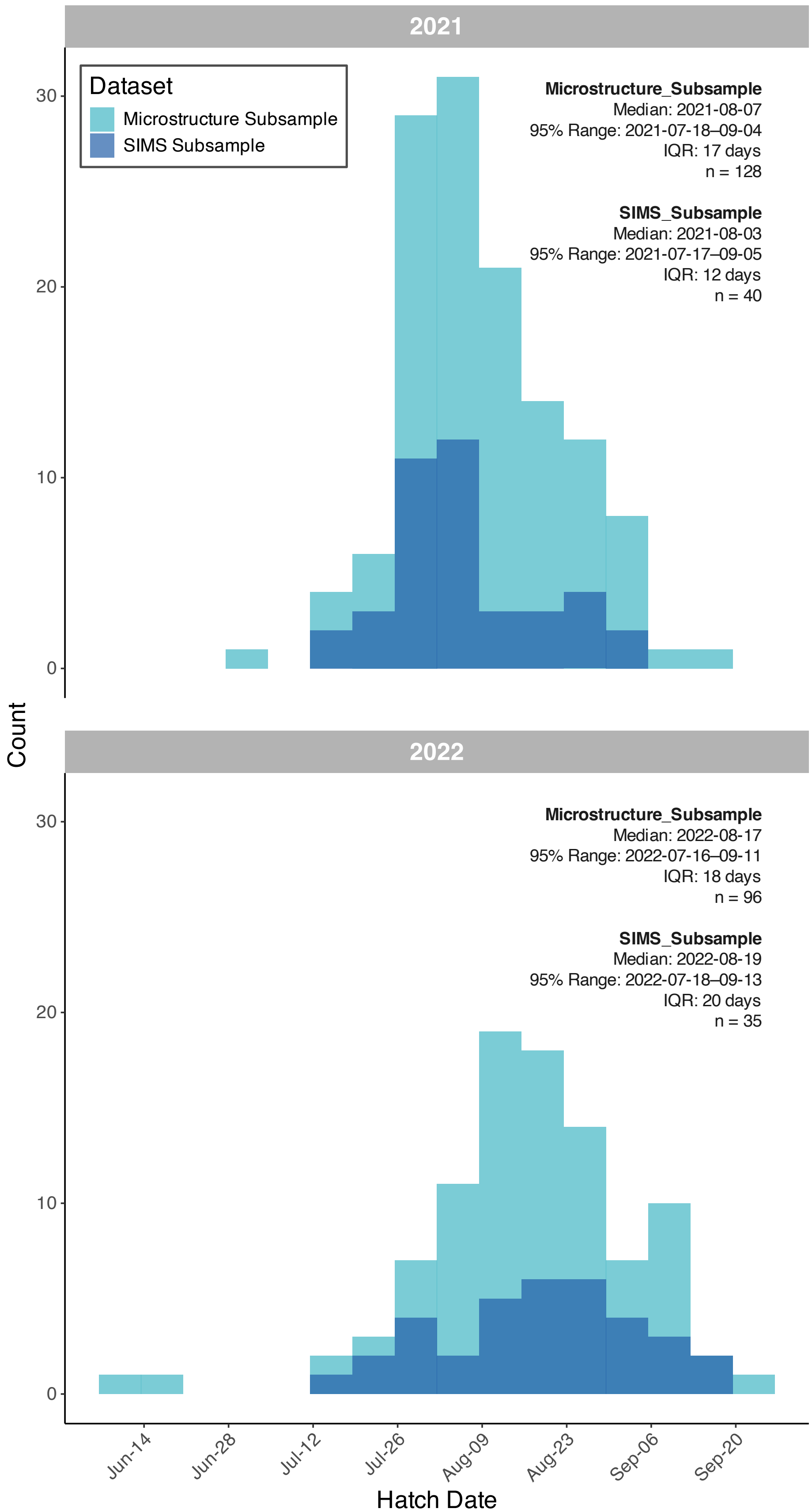
2021
Dataset
Microstructure Subsample
SIMS Subsample
Microstructure_Subsample
Median: 2021-08-07
95% Range: 2021-07-18–09-04
IQR: 17 days
n = 128
SIMS_Subsample
Median: 2021-08-03
95% Range: 2021-07-17–09-05
IQR: 12 days
n = 40
2022
Microstructure_Subsample
Median: 2022-08-17
95% Range: 2022-07-16–09-11
IQR: 18 days
n = 96
SIMS_Subsample
Median: 2022-08-19
95% Range: 2022-07-18–09-13
IQR: 20 days
n = 35
Count
0
10
20
30
Jun-14
Jun-28
Jul-12
Jul-26
Aug-09
Aug-23
Sep-06
Sep-20
Hatch Date

Fig. S2 | Otolith-derived hatch date distributions. Otolith-based hatch date distributions of juvenile Chinook salmon collected at the Red Bluff Diversion Dam in 2021 and 2022. Light blue indicates all individuals with estimated hatch dates, while dark blue highlights the subset of samples used for subsequent temperature reconstruction based on otolith stable oxygen isotope analysis using secondary ion mass spectrometry (SIMS). Summary statistics including the median, 95% range, interquartile range (IQR), and sample size are indicated for each group.

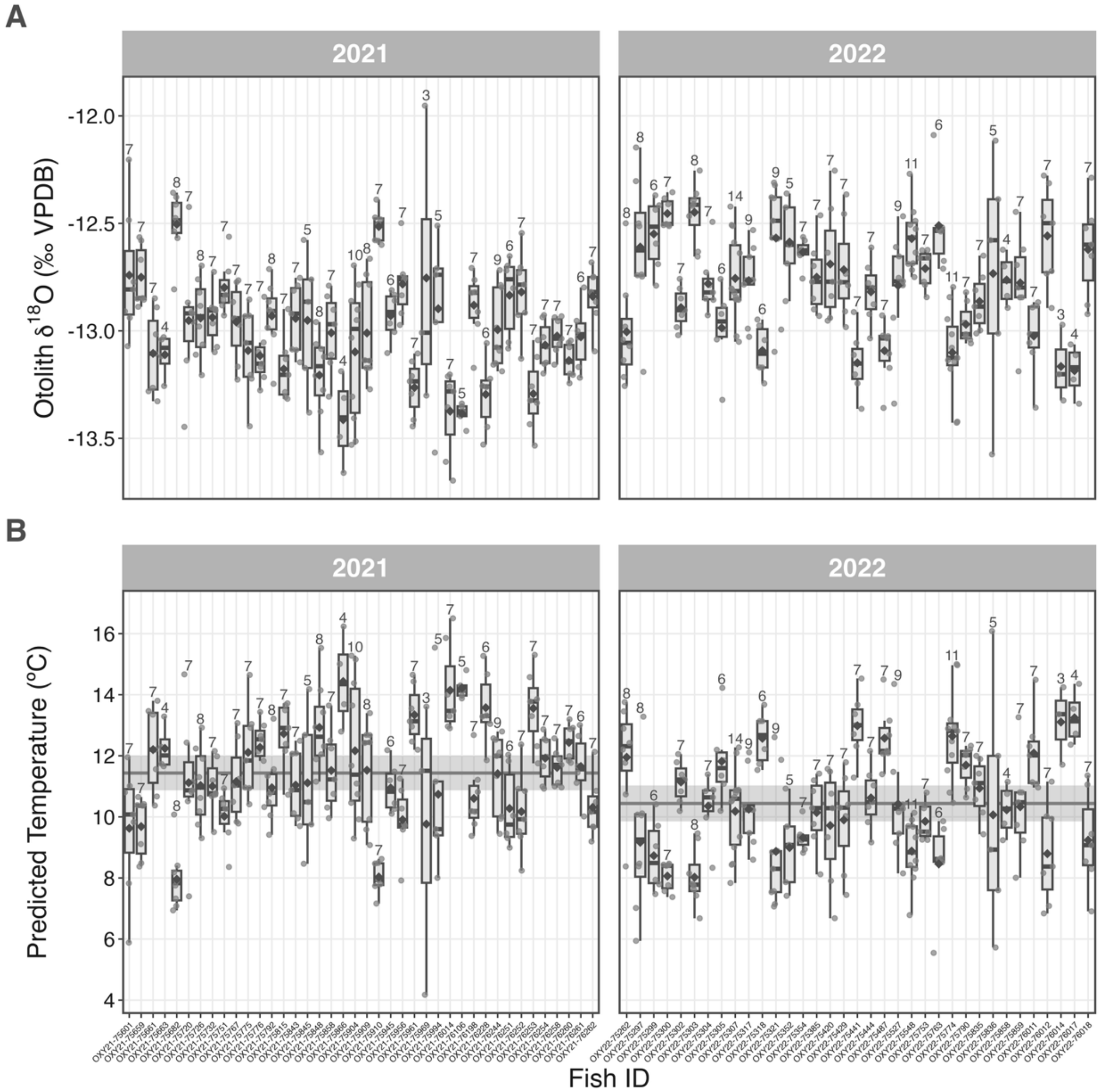
A
2021
2022
Otolith δ18O (‰ VPDB)
-12.0
-12.5
-13.0
-13.5
B
2021
2022
Predicted Temperature (°C)
16
14
12
10
8
6
4
Fish ID

Fig. S3 | Otolith $\delta^{18}O$ values and reconstructed pre-emergence temperatures. (**A**) Boxplots showing otolith stable isotope values ($\delta^{18}O$; VPDB scale) of juvenile Chinook salmon collected in 2021 (n = 40) and 2022 (n = 35), measured using secondary ion mass spectrometry (SIMS). (**B**) Corresponding reconstructed pre-emergence temperatures, derived from otolith $\delta^{18}O$ values, water $\delta^{18}O$ estimates, and isotope fractionation relationships. In both panels, solid diamonds represent individual mean values, while small open circles show individual SIMS measurements. Numbers above each boxplot indicate the number of SIMS measurements per fish. The solid line and shaded area in panel **B** represent the group mean predicted temperature and ±2 standard errors (SE), respectively.

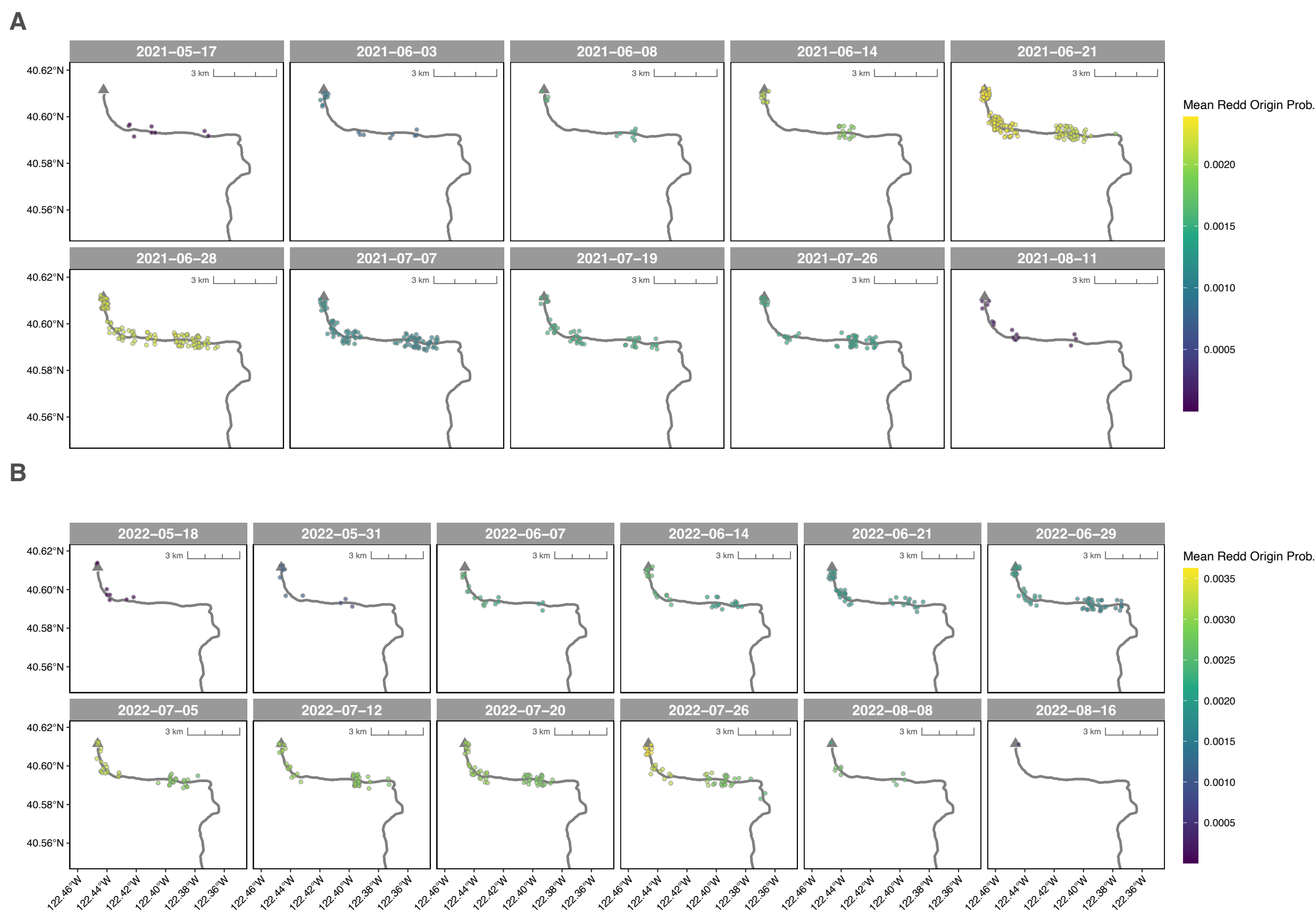

Fig. S4 | Population-level redd origin probabilities projected across the Sacramento River basin. Distribution of population-level mean redd origin probabilities assigned to individual redds observed throughout the Sacramento River basin. Each redd is plotted by its geographic location and colored by its mean probability of contributing to juvenile winter-run Chinook salmon survivors collected at the Red Bluff Diversion Dam in 2021 (**A**) and 2022 (**B**). Panels are grouped by redd deposition dates. A small random variation along the y-axis was added to each point to prevent overplotting.

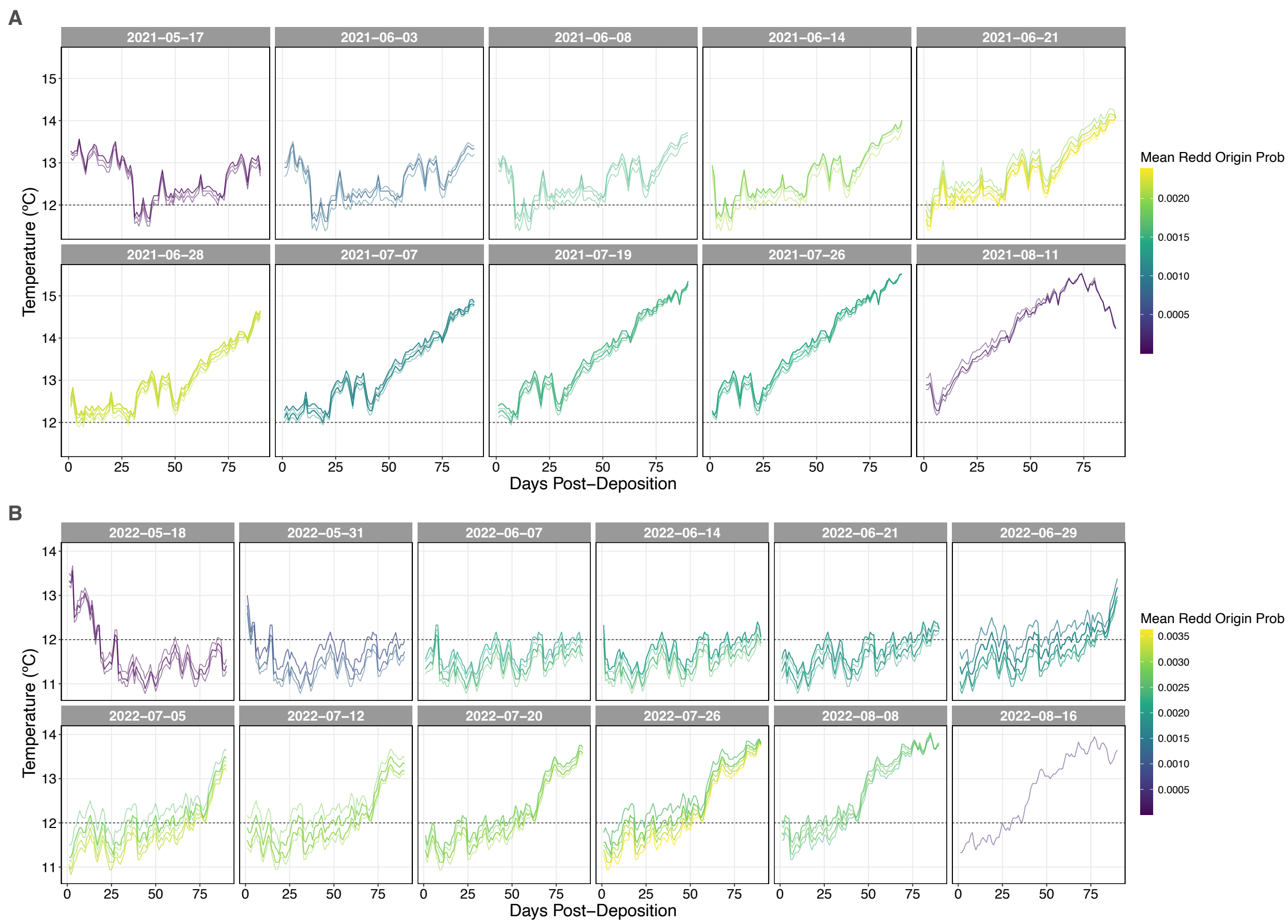

Fig. S5 | Modeled redd temperature profiles. Daily temperature profiles estimated from the RAFT (River Assessment for Forecasting Temperature) model for observed redd groups (redds sharing the same observation date and spatial location), colored by mean redd origin probability (i.e., inferred survival) for juvenile Chinook salmon survivors collected at the Red Bluff Diversion Dam in 2021 (**A**) and 2022 (**B**). Panels are separated by redd deposition dates. The dashed horizontal line denotes the 12 °C temperature threshold.

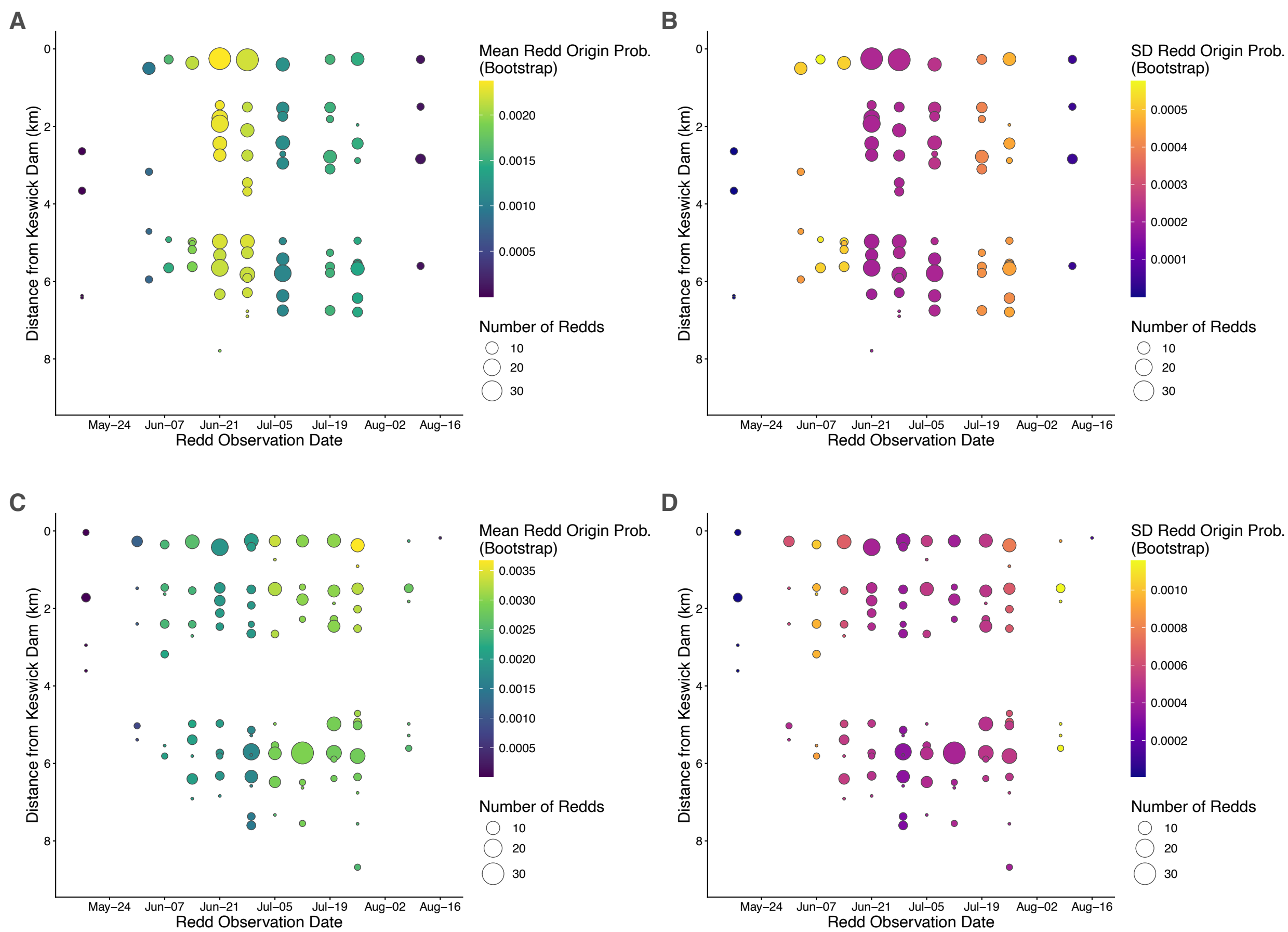

Fig. S6 | Bootstrapped estimates of population-level redd origin probabilities. Bootstrapped population-level mean (left panels **A** and **C**) and standard deviation (right panels **B** and **D**) of redd origin probabilities for juvenile winter-run Chinook salmon survivors collected at the Red Bluff Diversion Dam in 2021 (top row) and 2022 (bottom row). Redds are plotted by observation date (x-axis) and distance downstream from Keswick Dam (y-axis), with circle size proportional to the number of redds within each group.

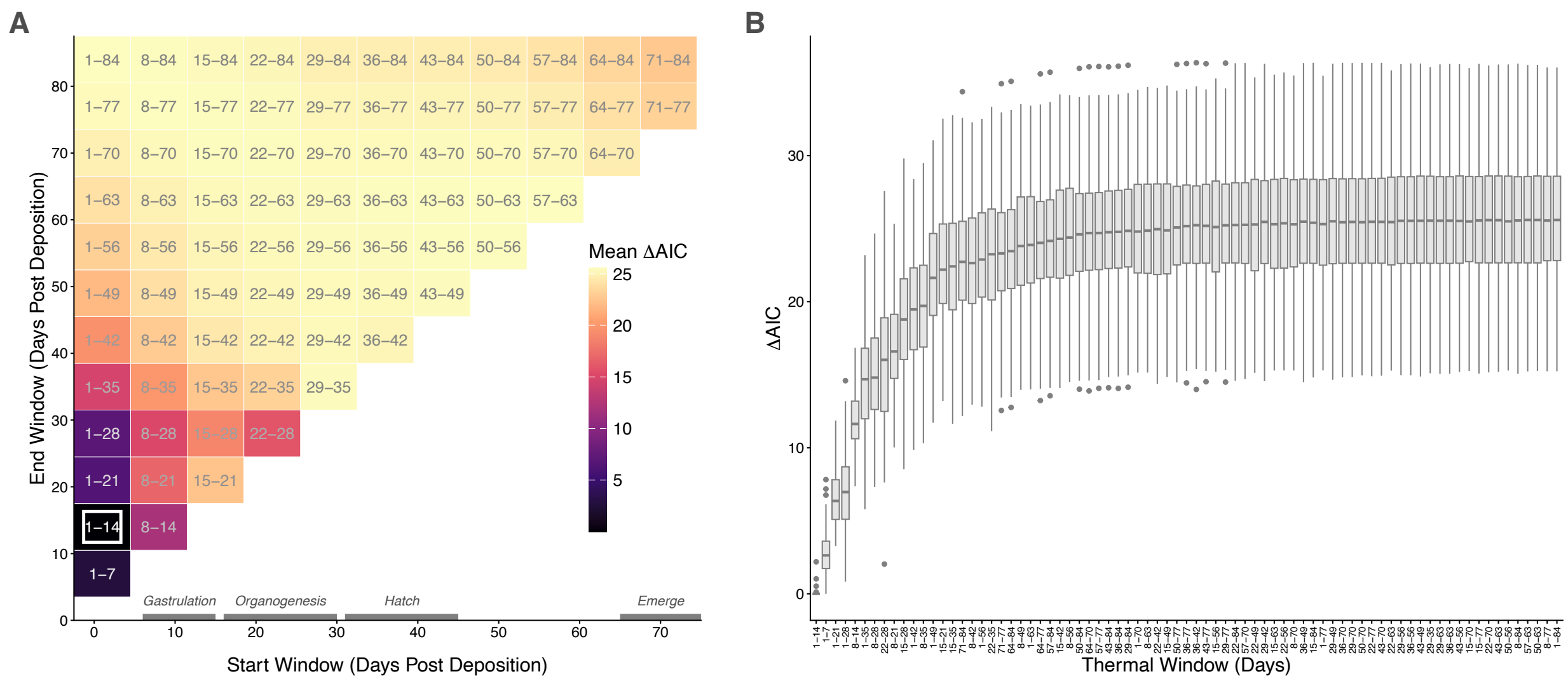

Fig. S7 | Bootstrapped estimates for the inferred critical thermal window. (**A**) Heatmap of mean ΔAIC (difference in Akaike Information Criterion) values from the moving window generalized linear model (GLM) analysis, repeated 100 times using bootstrapped sampling with 20% of redds held out in each iteration. Each grid cell corresponds to a specific combination of start and end days post-redd deposition, representing the temporal window over which mean temperature was calculated to predict the mean probability of redd origin. Lower ΔAIC values indicate better model fit. The white box highlights the optimal window (days 1–14 post-deposition), which consistently produced the lowest mean ΔAIC across bootstraps. Developmental stages and their approximate durations are shown along the x-axis. (**B**) Boxplots of ΔAIC values for each window combination, ranked by mean ΔAIC across the 100 bootstrap iterations, illustrating the relative stability and performance of each window.

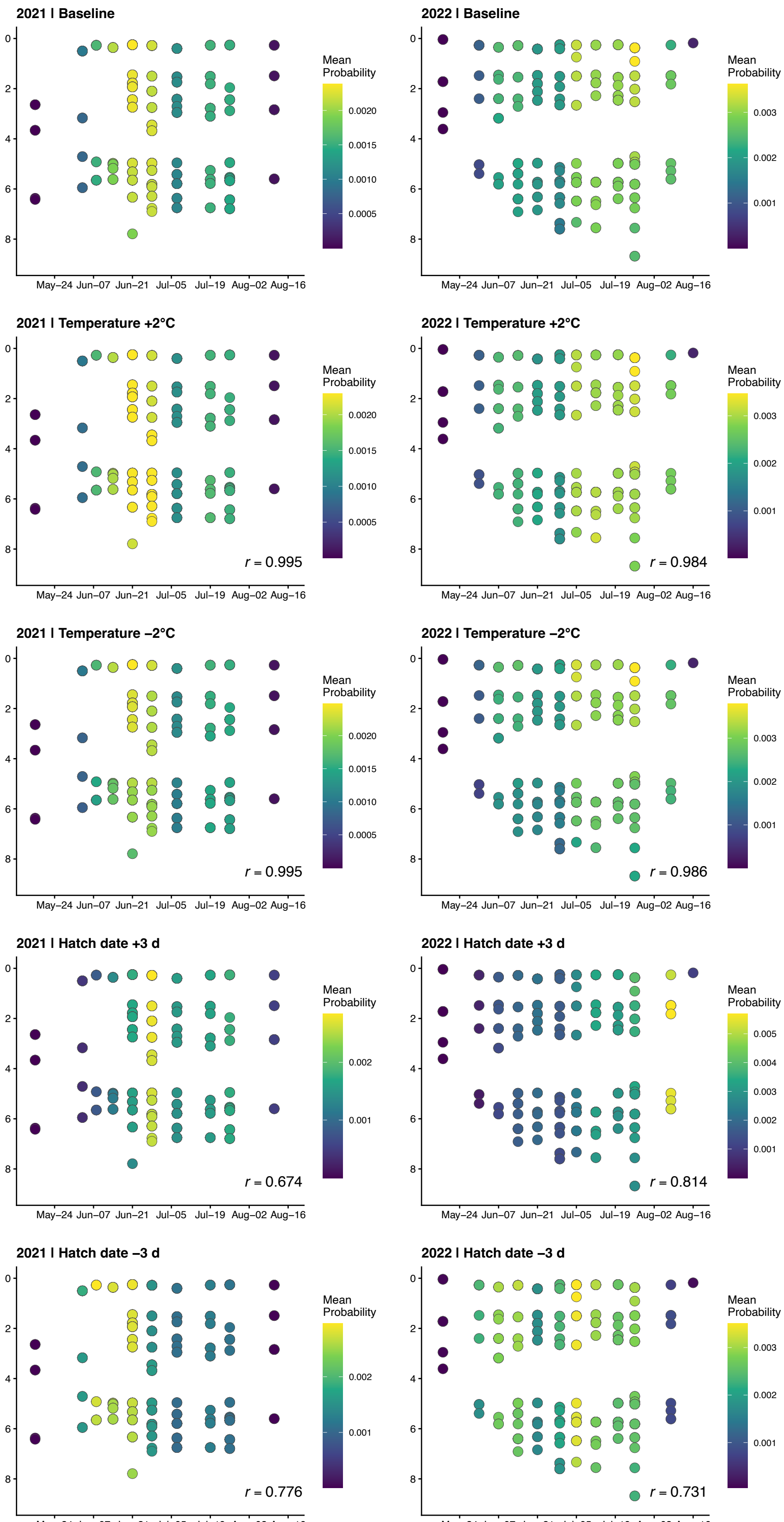
2021 | Baseline
2022 | Baseline
2021 | Temperature +2°C
2022 | Temperature +2°C
r = 0.995
r = 0.984
2021 | Temperature −2°C
2022 | Temperature −2°C
r = 0.995
r = 0.986
2021 | Hatch date +3 d
2022 | Hatch date +3 d
r = 0.674
r = 0.814
2021 | Hatch date −3 d
2022 | Hatch date −3 d
r = 0.776
r = 0.731
Mean Probability
0.0005
0.0010
0.0015
0.0020
0.001
0.002
0.003
0.004
0.005
0
2
4
6
8
May−24
Jun−07
Jun−21
Jul−05
Jul−19
Aug−02
Aug−16

Fig. S8 | Sensitivity of redd-origin assignments to temperature and hatch-date uncertainty. Perturbation analysis evaluating the sensitivity of inferred redd origin distributions for juvenile winter-run Chinook salmon to systematic shifts in reconstructed temperatures (±2 °C) and hatch dates (±3 d). Panels show spatial and temporal patterns of mean redd origin probabilities under the baseline scenario for 2021 and 2022, +2 °C temperature perturbations for 2021 and 2022, −2 °C temperature perturbations for 2021 and 2022, +3 d hatch-date perturbations for 2021 and 2022, and −3 d hatch-date perturbations for 2021 and 2022. Pearson correlation coefficients (r) comparing redd-origin probability surfaces across space and time relative to the baseline scenario are shown in the bottom-right corner of each perturbation panel.

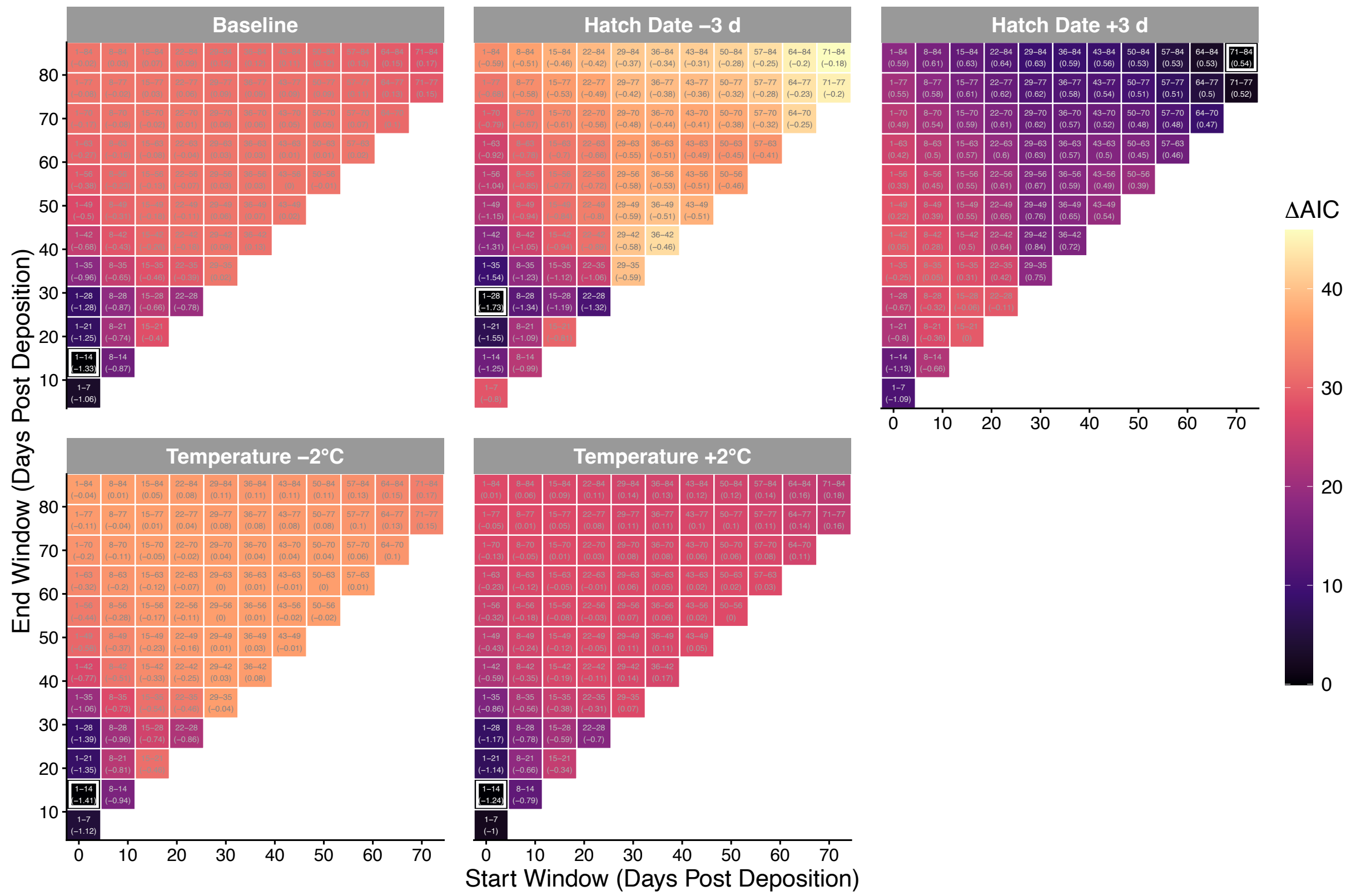

Fig. S9 | Sensitivity of critical thermal window to systematic perturbations. Perturbation analysis evaluating the sensitivity of the inferred critical thermal window for juvenile winter-run Chinook salmon to systematic shifts in reconstructed temperatures (±2°C) and hatch dates (±3 d). Heatmaps show ΔAIC (difference in Akaike Information Criterion) values from moving window generalized linear models (GLMs) fitted under each perturbation scenario (shown in strip labels). Each grid cell represents a unique start–end developmental window (days post deposition) used to calculate mean incubation temperature for predicting early survival. Values shown in parentheses within each cell indicate the estimated GLM temperature slope coefficient. The best-supported model within each scenario (ΔAIC = 0) is highlighted with a white box and interpreted as the inferred critical thermal window.

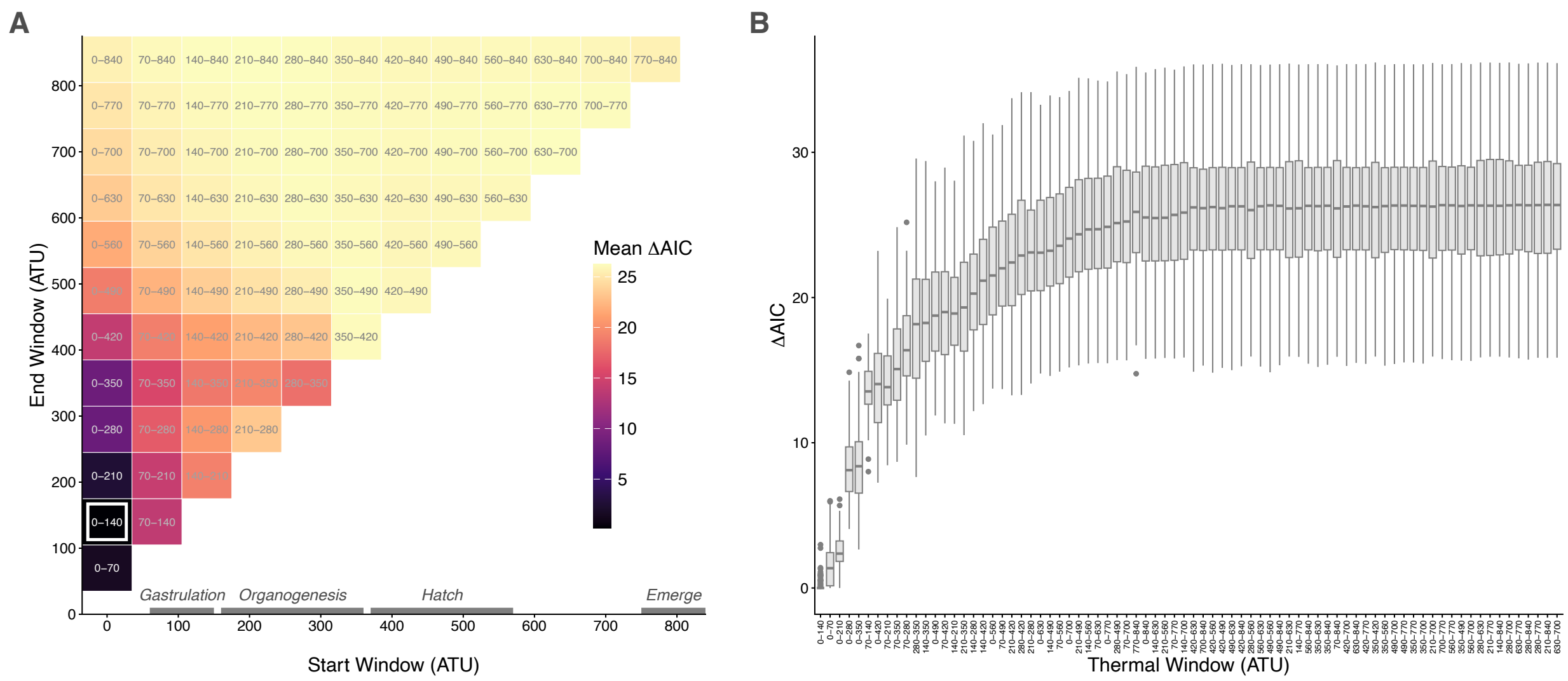

Fig. S10 | Bootstrapped estimates for the critical thermal window using accumulated temperature units. (**A**) Heatmap of mean ΔAIC (difference in Akaike Information Criterion) values from the moving window generalized linear model (GLM) analysis using accumulated temperature units (ATU), repeated 100 times using bootstrapped sampling with 20% of redds held out in each iteration. Each grid cell corresponds to a specific combination of start and end ATU, representing the temporal window over which mean temperature was calculated to predict the mean probability of redd origin. Lower ΔAIC values indicate better model fit. The white box highlights the optimal window (0–140 ATU), which consistently produced the lowest mean ΔAIC across bootstraps. Developmental stages and their approximate durations are shown along the x-axis. (**B**) Boxplots of ΔAIC values for each window combination, ranked by mean ΔAIC across the 100 bootstrap iterations, illustrating the relative stability and performance of each window.

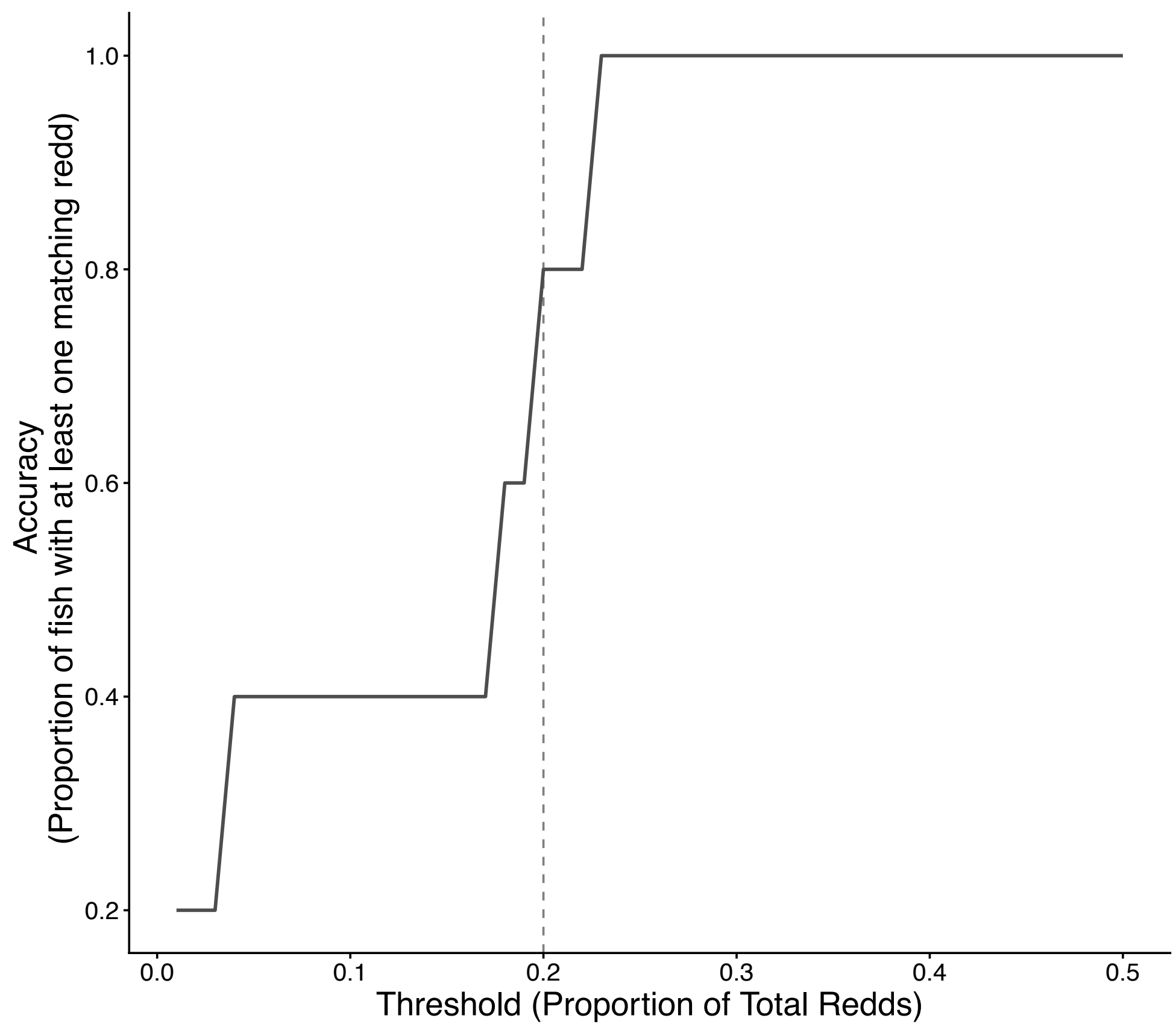

Fig. S11 | Trade-off between accuracy and precision in parentage-based tagging validation. Relationship between accuracy and precision in identifying matching redds between the otolith-informed and parentage-based tagging (PBT) probability models. Accuracy was defined as the proportion of fish with at least one overlapping "likely" redd between the two models, and precision was defined by the percentile threshold used to designate likely redds (i.e., upper 1–50% of redds). A higher threshold (more stringent precision) reduces the number of redds classified as likely. The vertical dashed line indicates the 0.2 threshold (80th percentile), which provided the most balanced trade-off between accuracy and precision and yielded matches for four of five fish (80% agreement).

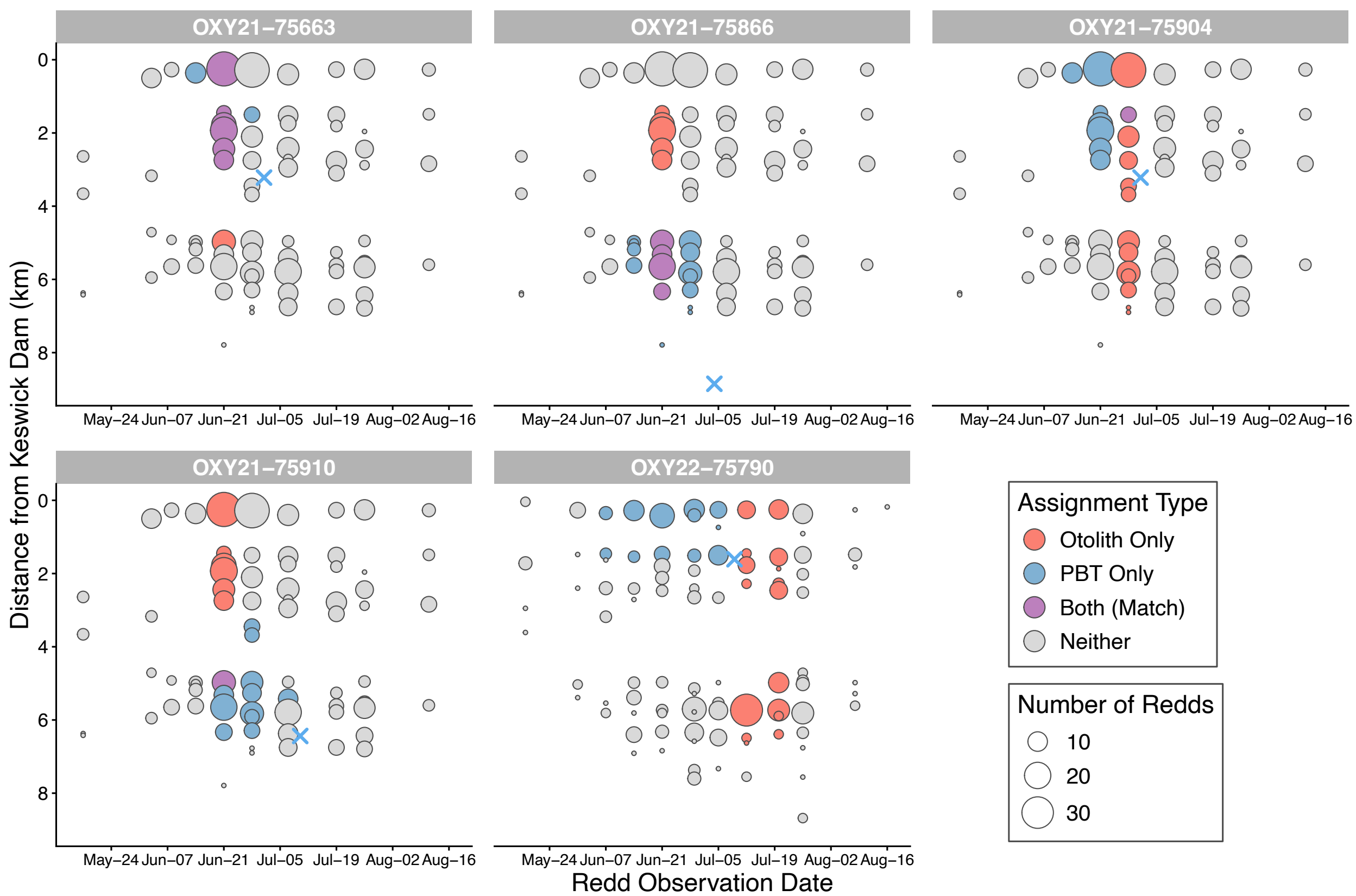

Fig. S12 | Agreement between otolith-informed and parentage-based redd assignments. Spatial and temporal distribution of redds showing overlap in "likely" redds (top 20%, or 80th percentile) between the otolith-informed and parentage-based tagging (PBT) probability models for five juveniles with known parents. Redds are color-coded to indicate those identified as matches (shared by both models), selected only by the PBT model, selected only by the otolith model, or not selected by either model. Blue X marks indicate the location and timing of the identified parent's carcass.

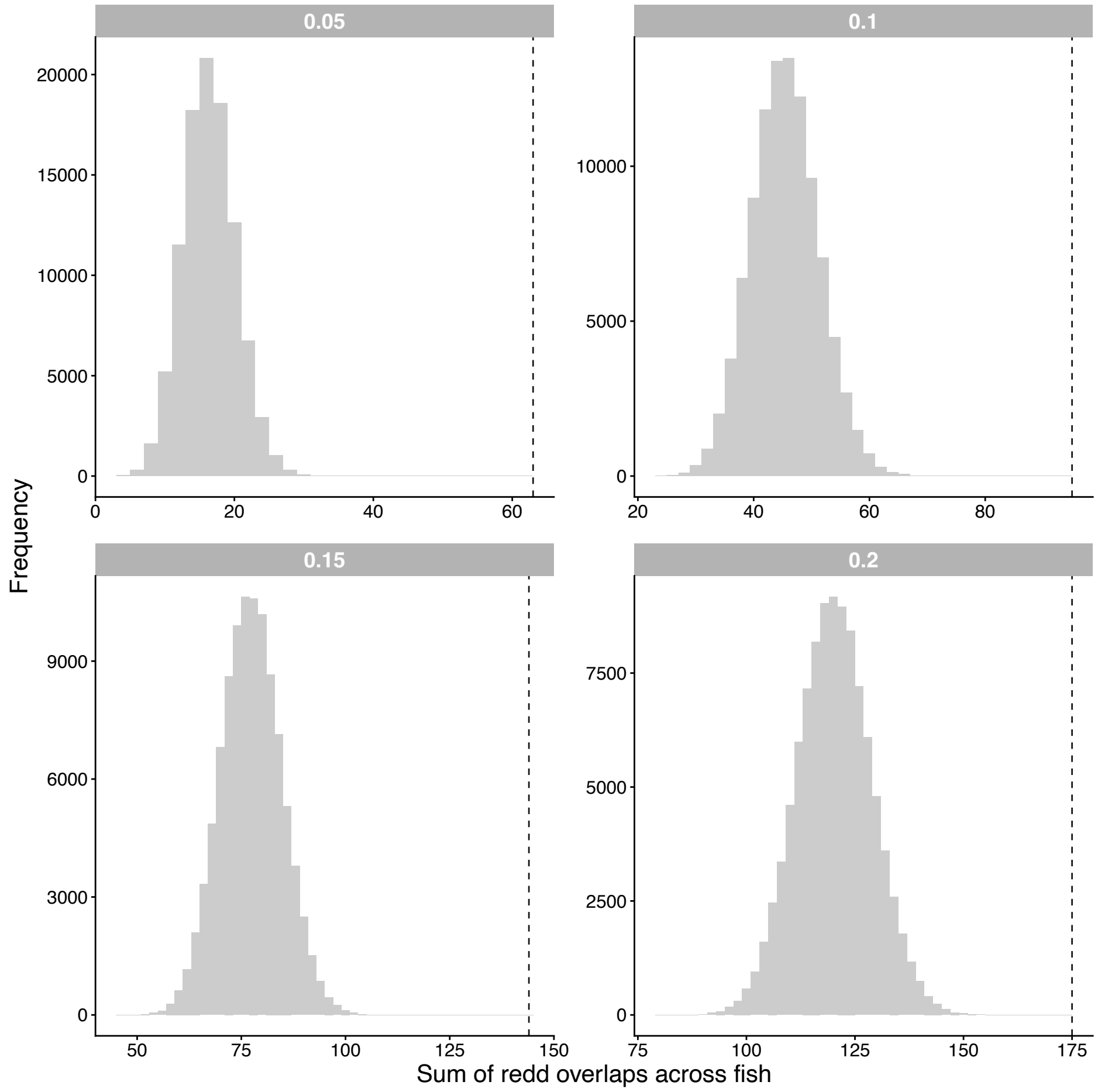

Fig. S13 | Statistical support for agreement between redd assignment models. Null distributions (gray) of the total number of overlapping redds across all fish compared with the observed totals (vertical dashed line) for each percentile threshold (0.05–0.2). Null expectations were generated from 100,000 random permutations of top-ranked redd sets while preserving the number of candidate redds per fish. Observed agreement between the otolith-informed and parentage-based tagging (PBT) models exceeded random expectations at all thresholds, indicating that the matches were unlikely to have occurred by chance.

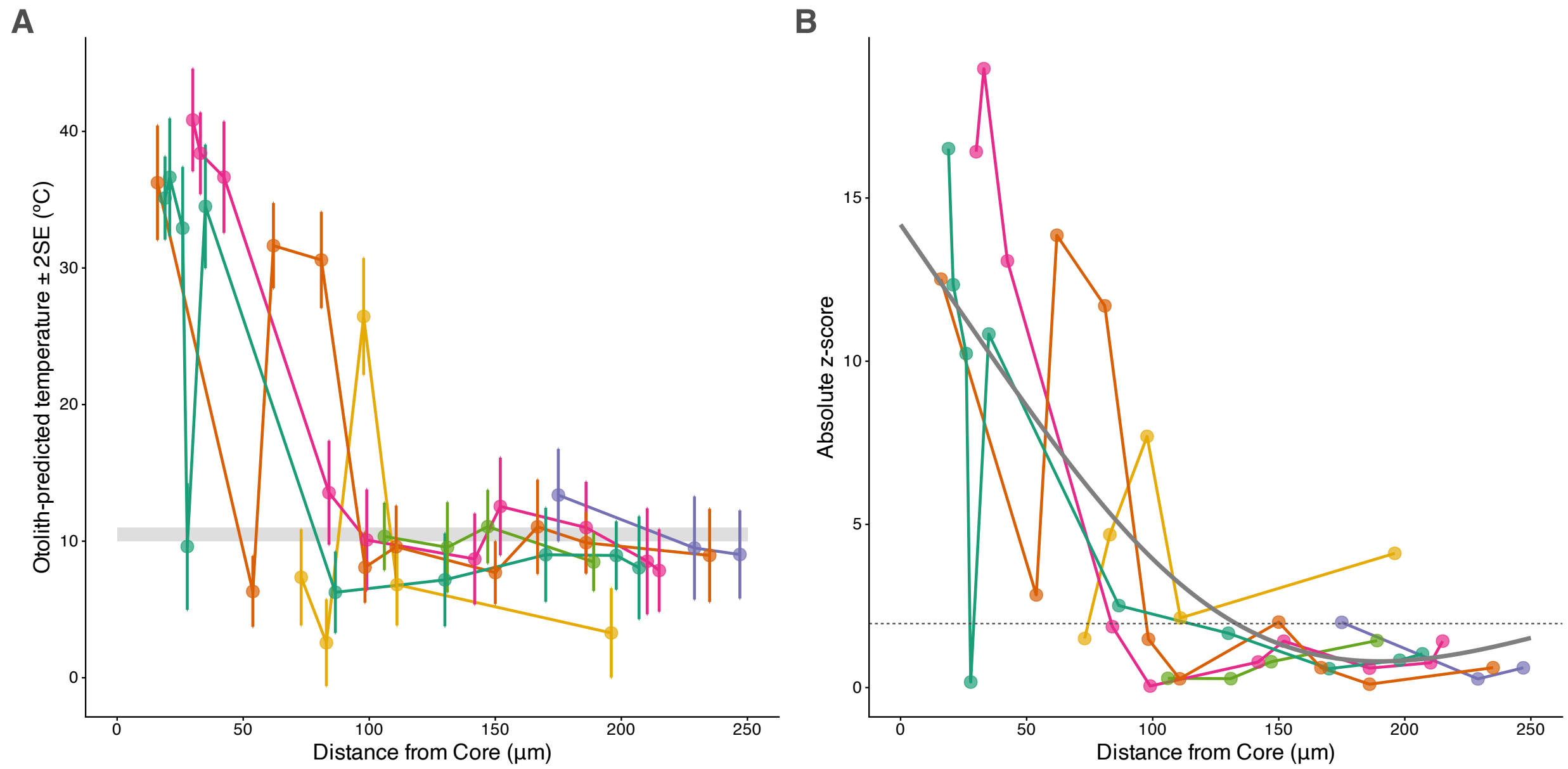

Fig. S14 | Validation of otolith-based temperature reconstructions under controlled conditions. (**A**) Reconstructed water temperature profiles (mean ± 2 SE) based on otolith $\delta^{18}O$ measurements via secondary ion mass spectrometry (SIMS) along transects from the core to the exogenous feeding check in tank-reared juvenile Chinook salmon ($n = 6$) held at constant, known temperatures. Colored lines represent individual temperature profiles. The gray shaded area indicates the observed range of tank temperatures (10.0–11.0 °C). (**B**) Absolute z-scores comparing reconstructed temperatures at each transect point to the known mean tank temperature (10 °C). The horizontal dashed line at 1.96 denotes the significance threshold ($\alpha = 0.05$). The gray curve shows a generalized additive model (GAM) fit to the absolute z-scores, illustrating the overall trend in temperature prediction accuracy with distance from the core.

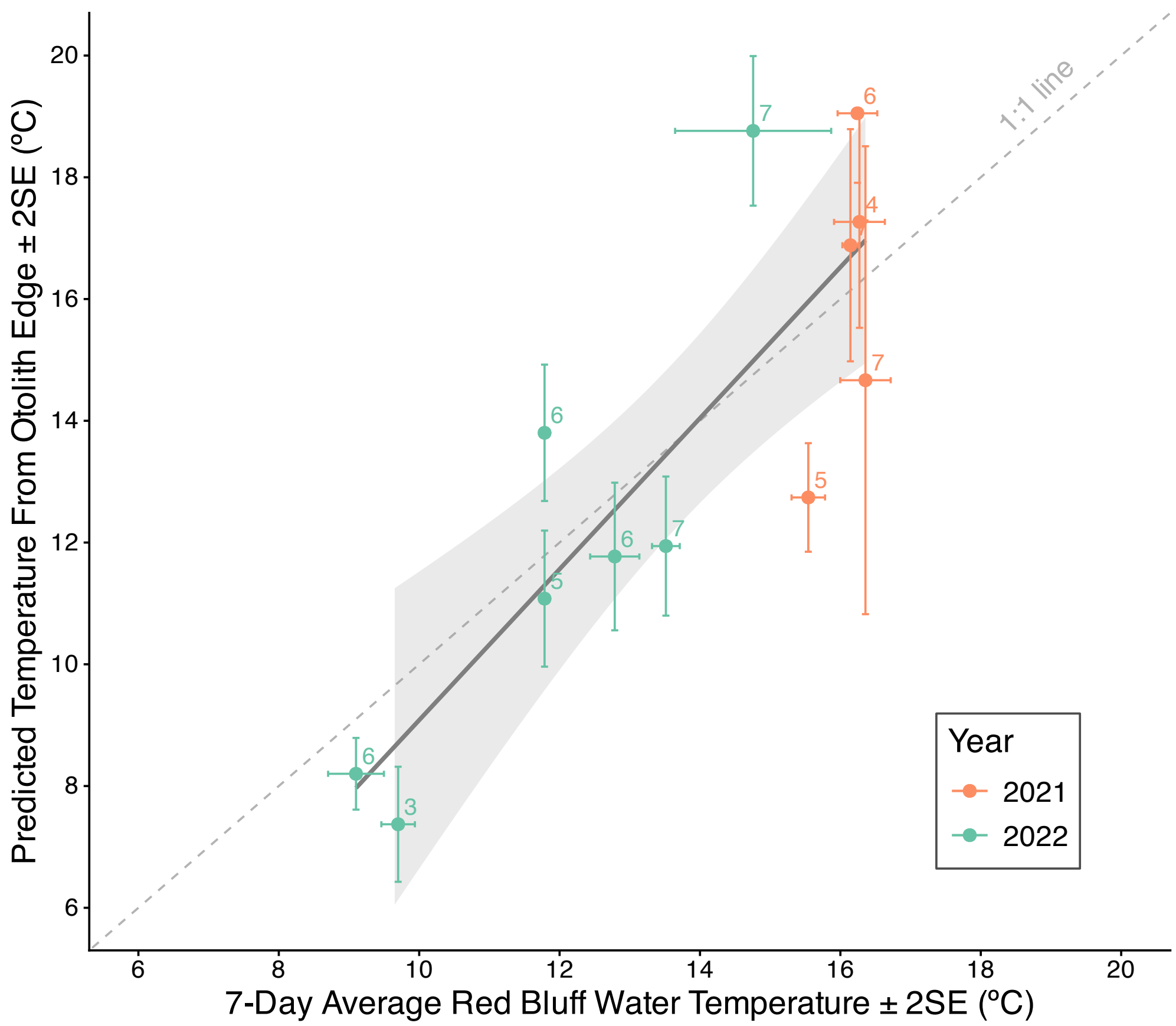

Fig. S15 | Otolith edge validation of otolith-based temperature reconstructions. Comparison of observed water temperatures at Red Bluff rotary screw trap at date of fish collection and predicted temperatures fish experienced close to date of collection. The x-axis shows the 7-days average water temperature at Red Bluff (the day of fish collection and six days prior). The y-axis shows the average predicted temperature from otolith oxygen isotope reconstructions (± 2 SE), using the secondary ion mass spectrometry (SIMS) spots closest to the otolith edge. Values were weighted by the number of days offset from the fish collection date, and otoliths with edge measurements >7 days from collection were excluded. The gray line represents the fitted linear regression (±95% CI), and the dashed gray line indicates the 1:1 line. Numbers above points denote the number of SIMS spots measured near the otolith edge.

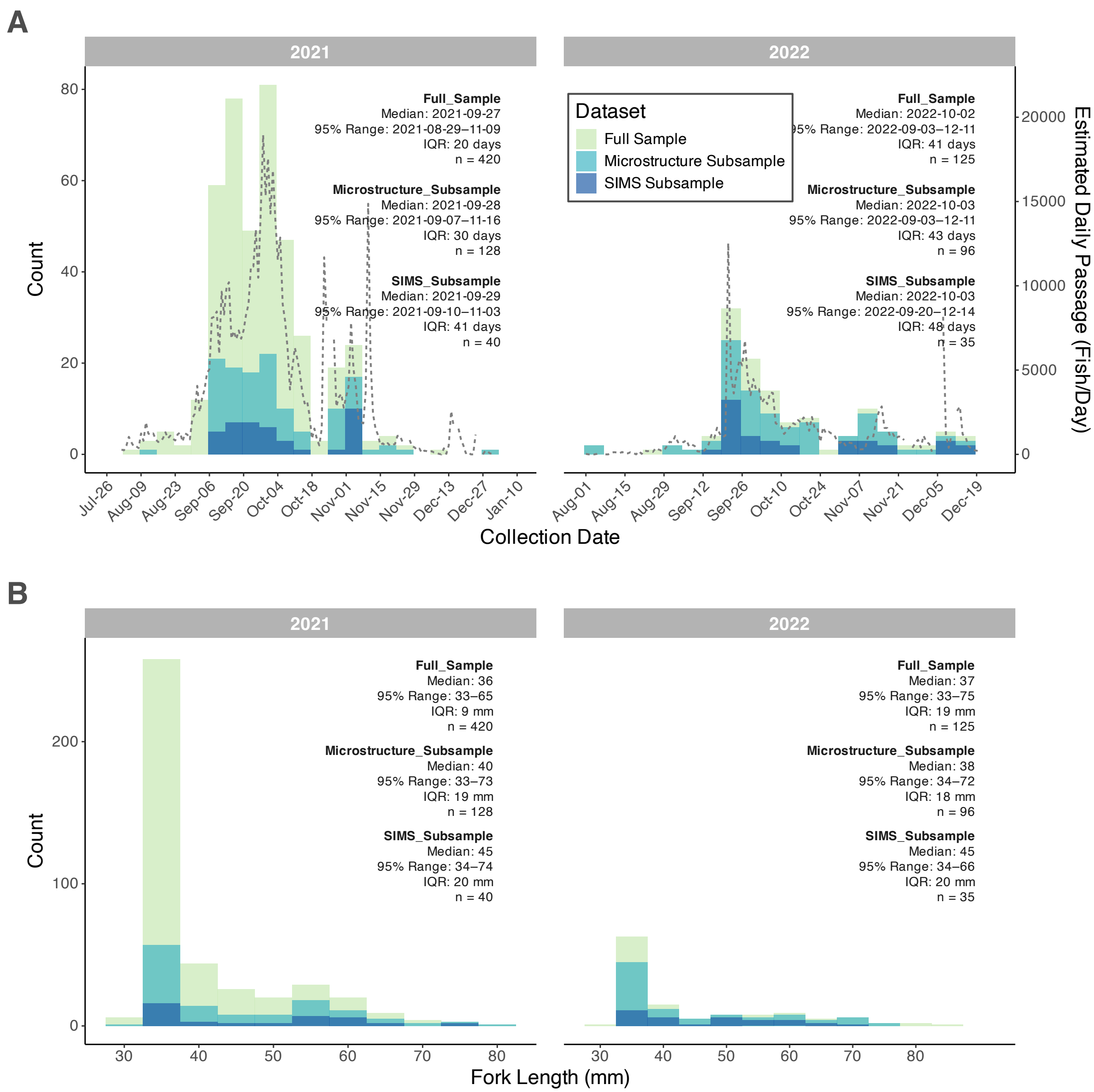
A
2021
2022
Full_Sample
Median: 2021-09-27
95% Range: 2021-08-29–11-09
IQR: 20 days
n = 420
Microstructure_Subsample
Median: 2021-09-28
95% Range: 2021-09-07–11-16
IQR: 30 days
n = 128
SIMS_Subsample
Median: 2021-09-29
95% Range: 2021-09-10–11-03
IQR: 41 days
n = 40
Full_Sample
Median: 2022-10-02
95% Range: 2022-09-03–12-11
IQR: 41 days
n = 125
Microstructure_Subsample
Median: 2022-10-03
95% Range: 2022-09-03–12-11
IQR: 43 days
n = 96
SIMS_Subsample
Median: 2022-10-03
95% Range: 2022-09-20–12-14
IQR: 48 days
n = 35
Dataset
Full Sample
Microstructure Subsample
SIMS Subsample
Count
Estimated Daily Passage (Fish/Day)
Collection Date
B
2021
2022
Full_Sample
Median: 36
95% Range: 33–65
IQR: 9 mm
n = 420
Microstructure_Subsample
Median: 40
95% Range: 33–73
IQR: 19 mm
n = 128
SIMS_Subsample
Median: 45
95% Range: 34–74
IQR: 20 mm
n = 40
Full_Sample
Median: 37
95% Range: 33–75
IQR: 19 mm
n = 125
Microstructure_Subsample
Median: 38
95% Range: 34–72
IQR: 18 mm
n = 96
SIMS_Subsample
Median: 45
95% Range: 34–66
IQR: 20 mm
n = 35
Count
Fork Length (mm)

Fig. S16 | Characteristics of juvenile Chinook salmon included in otolith analyses. Histograms showing (**A**) collection dates and (**B**) fork lengths of winter-run juvenile Chinook salmon sampled at the Red Bluff Diversion Dam in 2021 and 2022. Bars represent all incidental mortalities collected, along with the subsets selected for otolith microstructure analysis and stable oxygen isotope analysis using secondary ion mass spectrometry (SIMS). Dashed lines in panel (**A**) indicate daily fish passage estimates from the SacPAS database (www.cbr.washington.edu/sacramento/). Summary statistics including median, 95% range, interquartile range (IQR), and sample size are provided for each group.

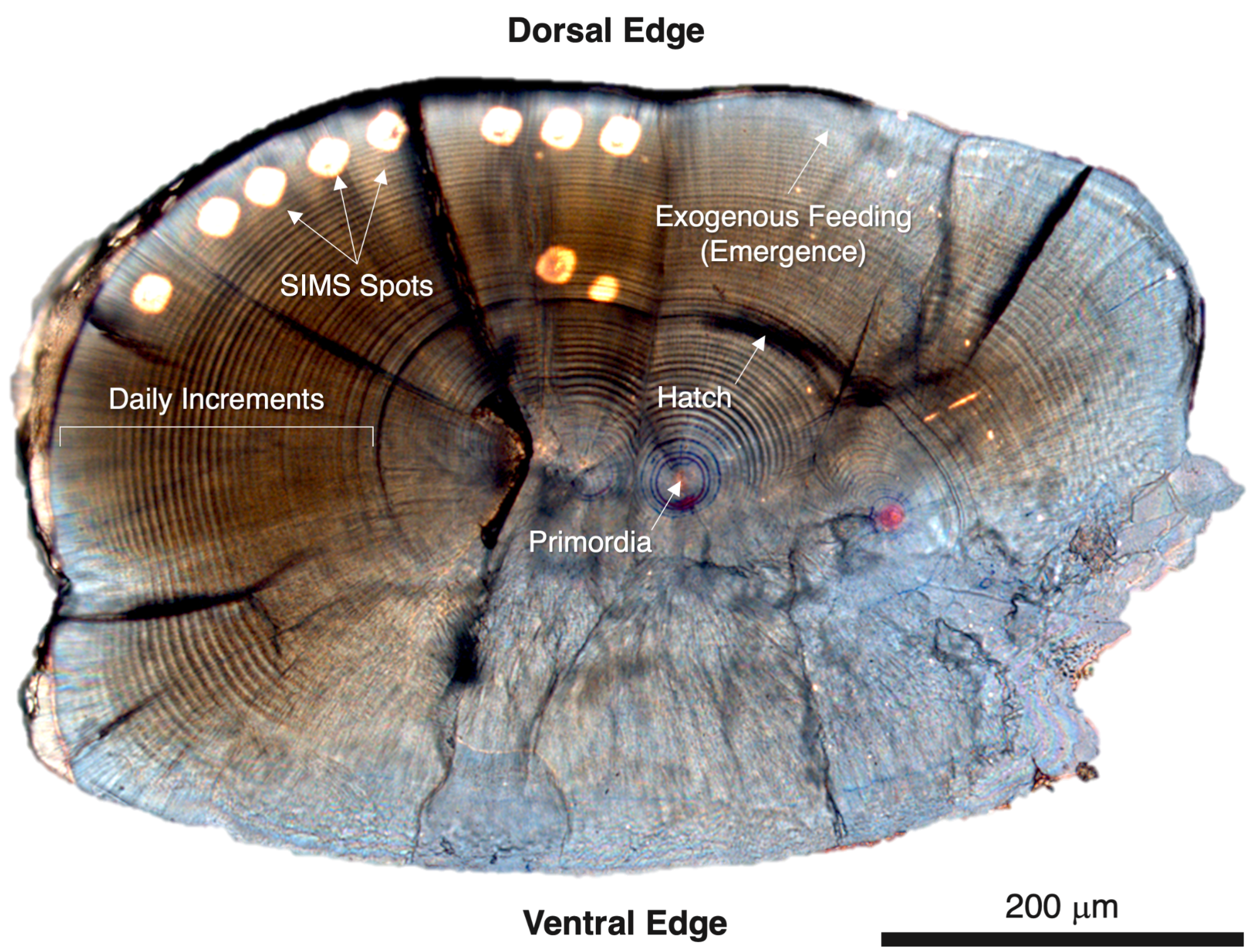

Fig. S17 | Otolith microstructure features used for age and temperature reconstruction. The primordia, hatch check, exogenous feeding check (corresponding to fry emergence from redds), and daily increments are annotated. The location of a secondary ion mass spectrometry (SIMS) spot sputtered immediately prior to the exogenous feeding check is also shown.

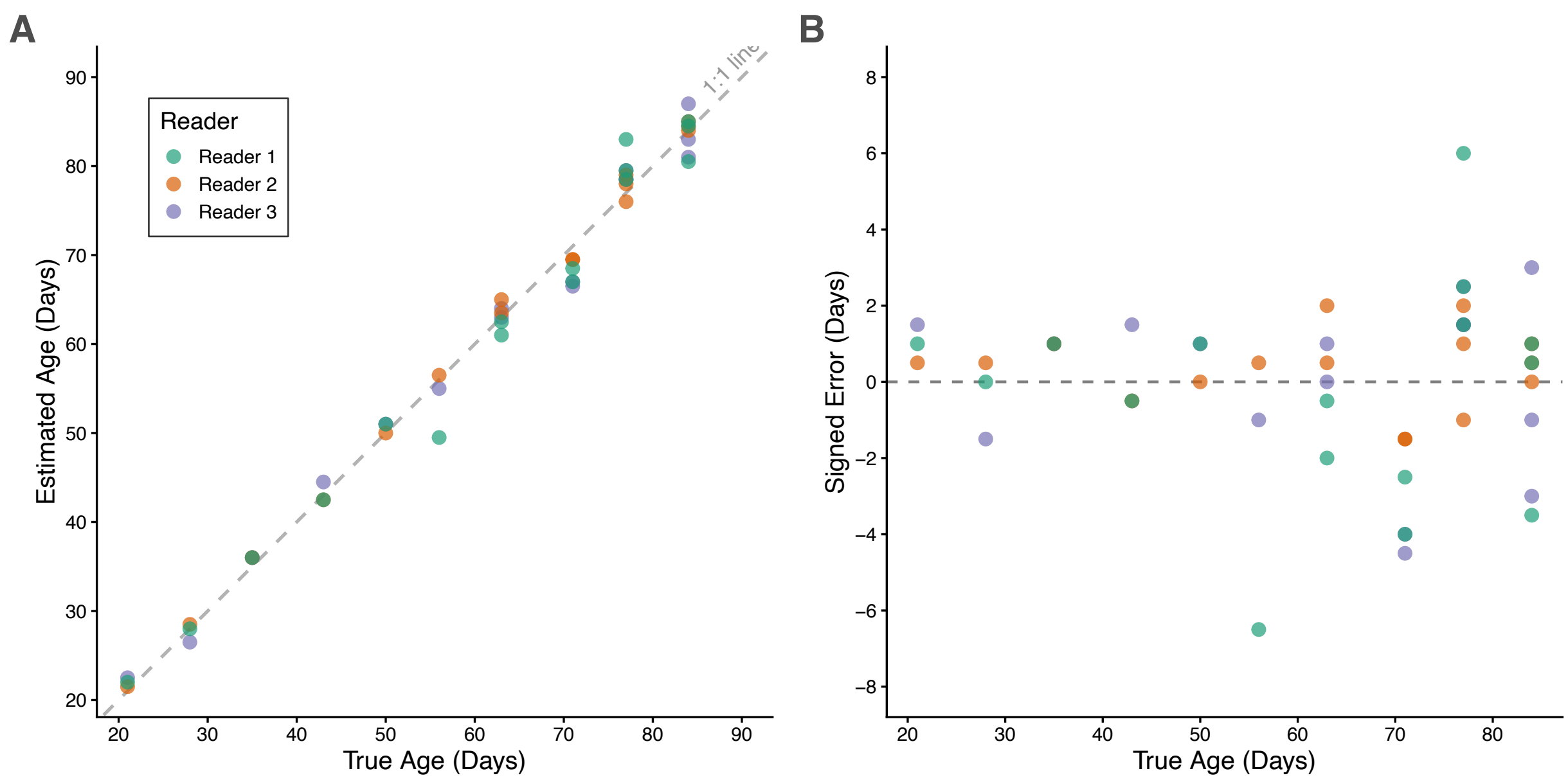

Fig. S18 | Validation of otolith-based age estimation using known-age fish. Validation of otolith-based age estimation using known-age juvenile Chinook salmon from Coleman National Fish Hatchery (*n* = 16). (**A**) Comparison between true age and estimated age derived from otolith microstructure analysis. Points represent the mean age estimate from two replicate reads conducted by three independent readers. The gray dashed line indicates the 1:1 relationship between true and estimated age. (**B**) Signed error (estimated age − true age) plotted against true age. Positive values indicate overestimation and negative values indicate underestimation of age. The horizontal gray dashed line indicates zero error.

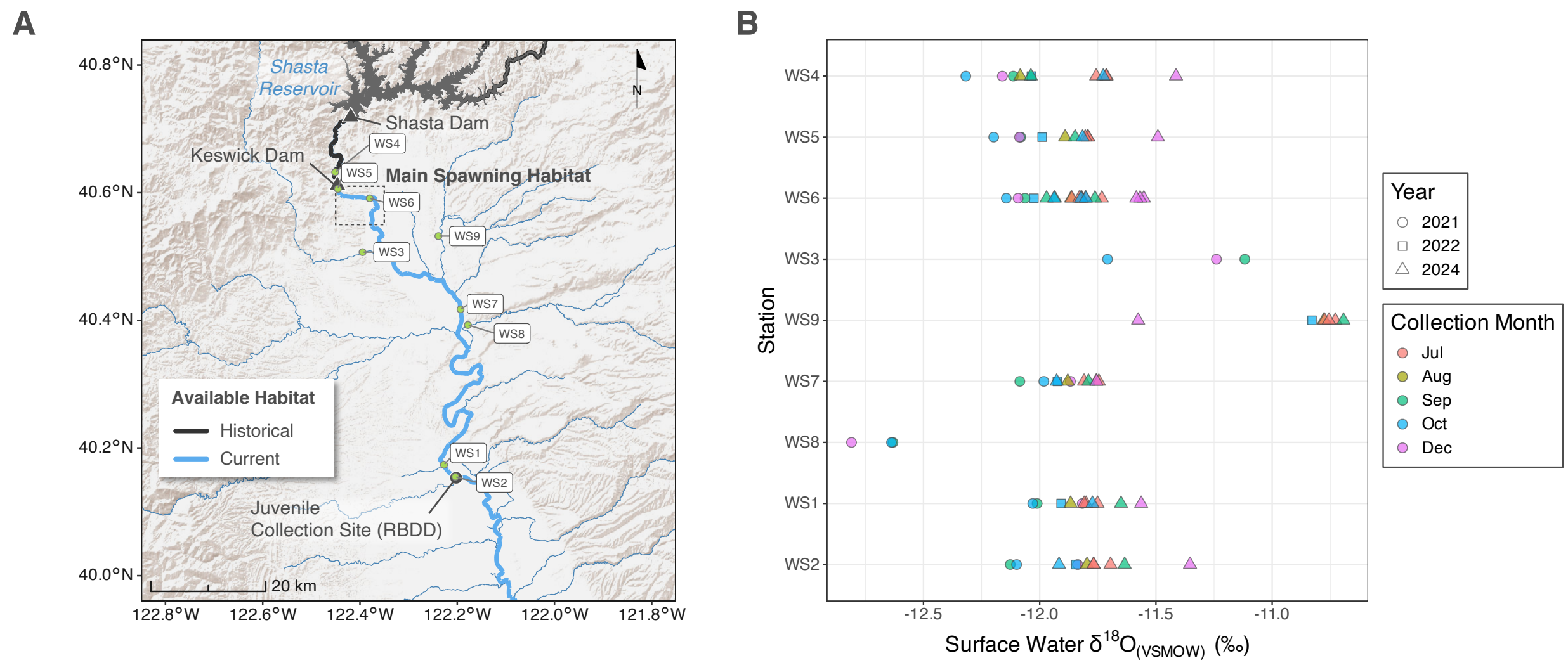

Fig. S19 | Spatial and temporal variation in river water δ^{18}O values. (**A**) Map of the Sacramento River showing water stable oxygen isotope (δ^{18}O) sampling sites (green circles, WS1–9) from 2021, 2022, and 2024. Also shown are historical (black) and current (blue) habitats available to Central Valley winter-run Chinook salmon, major dams (triangles), the juvenile collection site at the Red Bluff Diversion Dam (solid circle, RBDD), and the primary spawning habitat (dashed rectangle) below Keswick Dam. (**B**) Surface water $\delta^{18}O_{(VSMOW)}$ values by station and sampling date from September–December 2021, October 2022, and July–October and December 2024.

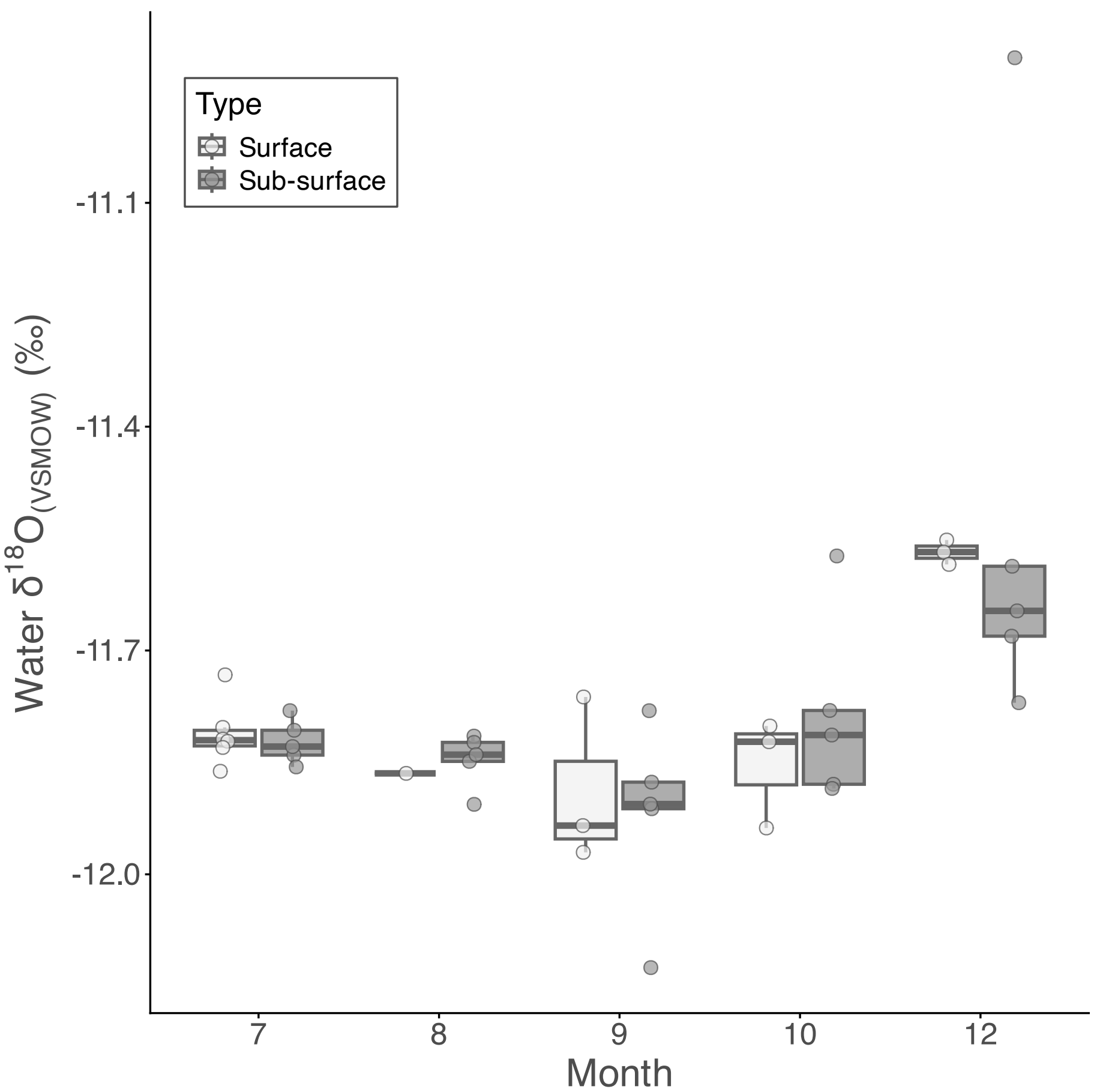

Fig. S20 | Surface and subsurface water $\delta^{18}O$ dynamics near spawning habitat. Surface and subsurface water oxygen isotope values ($\delta^{18}O$) from the mainstem Sacramento River at site WS6, near the primary winter-run Chinook salmon spawning habitat, collected between July–October and December 2024.

REFERENCES

1. A. J. Reid, A. K. Carlson, I. F. Creed, E. J. Eliason, P. A. Gell, P. T. J. Johnson, K. A. Kidd, T. J. MacCormack, J. D. Olden, S. J. Ormerod, J. P. Smol, W. W. Taylor, K. Tockner, J. C. Vermaire, D. Dudgeon, S. J. Cooke, Emerging threats and persistent conservation challenges for freshwater biodiversity. *Biol. Rev.* **94**, 849–873 (2019).

2. L. Comte, J. D. Olden, Climatic vulnerability of the world's freshwater and marine fishes. *Nat. Clim. Change* **7**, 718–722 (2017).

3. V. Barbarossa, J. Bosmans, N. Wanders, H. King, M. F. P. Bierkens, M. A. J. Huijbregts, A. M. Schipper, Threats of global warming to the world's freshwater fishes. *Nat. Commun.* **12**, 1701 (2021).

4. H. O. Pörtner, M. A. Peck, Climate change effects on fishes and fisheries: Towards a cause-and-effect understanding. *J. Fish Biol.* **77**, 1745–1779 (2010).

5. I.-C. Chen, J. K. Hill, R. Ohlemüller, D. B. Roy, C. D. Thomas, Rapid range shifts of species associated with high levels of climate warming. *Science* **333**, 1024–1026 (2011).

6. M. L. Pinsky, R. L. Selden, Z. J. Kitchel, Climate-driven shifts in marine species ranges: Scaling from organisms to communities. *Ann. Rev. Mar. Sci.* **12**, 153–179 (2020).

7. J. D. Olden, R. J. Naiman, Incorporating thermal regimes into environmental flows assessments: Modifying dam operations to restore freshwater ecosystem integrity. *Freshw. Biol.* **55**, 86–107 (2010).

8. G. Woodward, D. M. Perkins, L. E. Brown, Climate change and freshwater ecosystems: Impacts across multiple levels of organization. *Philos. Trans. R. Soc. B* **365**, 2093–2106 (2010).

9. F. T. Dahlke, S. Wohlrab, M. Butzin, H.-O. Pörtner, Thermal bottlenecks in the life cycle define climate vulnerability of fish. *Science* **369**, 65–70 (2020).

10. H. O. Pörtner, A. P. Farrell, Physiology and climate change. *Science* **322**, 690–692 (2008).

11. D. H. Secor, *Migration Ecology of Marine Fishes* (Johns Hopkins Univ. Press, Baltimore (Md.), 2015).

12. D. H. Secor, E. D. Houde, Temperature effects on the timing of striped bass egg production, larval viability, and recruitment potential in the Patuxent River (Chesapeake Bay). *Estuaries* **18**, 527–544 (1995).

13. A. M. Sturrock, S. M. Carlson, J. D. Wikert, T. Heyne, S. Nusslé, J. E. Merz, H. J. W. Sturrock, R. C. Johnson, Unnatural selection of salmon life histories in a modified riverscape. *Glob. Change Biol.* **26**, 1235–1247 (2020).

14. A. M. Sturrock, K. Sellheim, J. Merz, J. Sweeney, M. Bell-Tilcock, G. Whitman, K. Arai, M. Willmes, C. Jeffres, R. C. Johnson, A modern ghost story: Increased selective mortality of salmon under climate extremes. *Glob. Change Biol.* **32**, e70854 (2026).

15. B. T. Martin, A. Pike, S. N. John, N. Hamda, J. Roberts, S. T. Lindley, E. M. Danner, Phenomenological vs. Biophysical models of thermal stress in aquatic eggs. *Ecol. Lett.* **20**, 50–59 (2017).

16. J. J. Anderson, W. N. Beer, J. A. Israel, S. Greene, Targeting river operations to the critical thermal window of fish incubation: Model and case study on Sacramento River winter-run Chinook salmon. *River Res. Appl.* **38**, 895–905 (2022).

17. J. Bloomer, J. J. Anderson, D. Sear, S. Greene, D. Gantner, C. Hanson, Gastrulation and hatch as critical thermal windows for salmonid embryo development. *River Res. Appl.* **39**, 46–53 (2023).

18. B. T. Martin, P. N. Dudley, N. S. Kashef, D. M. Stafford, W. J. Reeder, D. Tonina, A. M. Del Rio, J. Scott Foott, E. M. Danner, The biophysical basis of thermal tolerance in fish eggs. *Proc. R. Soc. B* **287**, 20201550 (2020).

19. R. M. Yoshiyama, F. W. Fisher, P. B. Moyle, Historical abundance and decline of Chinook salmon in the Central Valley region of California. *N. Am. J. Fish. Manag.* **18**, 487–521 (1998).

20. J. G. Williams, Central Valley salmon: A perspective on Chinook and steelhead in the Central Valley of California (San Francisco Estuary Watershed Science, 2006), vol. 4. 10.15447/sfews.2006v4iss3art2.

21. R. C. Johnson, S. Windell, P. L. Brandes, J. L. Conrad, J. Ferguson, P. A. L. Goertler, B. N. Harvey, J. Heublein, J. A. Israel, D. W. Kratville, J. E. Kirsch, R. W. Perry, J. Pisciotto, W. R. Poytress, K. Reece, B. G. Swart, Science advancements key to increasing management value of life stage monitoring networks for endangered Sacramento River winter-run Chinook salmon in California (San Francisco Estuary Watershed. Science, 2017), vol. 15. 10.15447/sfews.2017v15iss3art1.

22. P. Reis-Santos, B. M. Gillanders, A. M. Sturrock, C. Izzo, D. S. Oxman, J. A. Lueders-Dumont, K. Hüssy, S. E. Tanner, T. Rogers, Z. A. Doubleday, A. H. Andrews, C. Trueman, D. Brophy, J. D. Thiem, L. J. Baumgartner, M. Willmes, M.-T. Chung, P. Charapata, R. C. Johnson, S. Trumble, Y. Heimbrand, K. E. Limburg, B. D. Walther, Reading the biomineralized book of life: Expanding otolith biogeochemical research and applications for fisheries and ecosystem-based management. *Rev. Fish Biol. Fish.* **33**, 411–449 (2023).

23. O. Morissette, C. N. Trueman, A. M. Sturrock, A. J. Geffen, K. Shirai, Limited evidence for species-specific sensitivity of temperature-dependent fractionation of oxygen stable isotope in biominerals: A meta-analysis. *Methods Ecol. Evol.* **14**, 1719–1731 (2023).

24. S. R. Thorrold, S. E. Campana, C. M. Jones, P. K. Swart, Factors determining $\delta^{13}C$ and $\delta^{18}O$ fractionation in aragonitic otoliths of marine fish. *Geochim. Cosmochim. Acta* **61**, 2909–2919 (1997).

25. K. Arai, M. Willmes, G. E. Whitman, K. W. Zillig, C. J. Cooper, N. Matsuda, E. A. Bell, K. D. McKeegan, L. S. Lewis, C. A. Jeffres, R. C. Johnson, Inter-method differences (SIMS vs. IRMS) in oxygen isotope fractionation: Insights from Chinook salmon otoliths. *Geochim. Cosmochim. Acta* **411**, 161–172 (2025).

26. T. Sakamoto, Simple visualization of fish migration history based on high-resolution otolith $\delta^{18}O$ profiles and hydrodynamic models. *Limnol. Oceanogr. Lett.* **10**, 101–112 (2025).

27. O. Morissette, L. Bernatchez, M. Wiedenbeck, P. Sirois, Deciphering lifelong thermal niche using otolith δ18O thermometry within supplemented lake trout (*Salvelinus namaycush*) populations. *Freshw. Biol.* **65**, 1114–1127 (2020).

28. S. T. Lindley, R. S. Schick, E. Mora, P. B. Adams, J. J. Anderson, S. Greene, C. Hanson, B. P. May, D. McEwan, R. B. MacFarlane, C. Swanson, J. G. Williams, Framework for assessing viability of threatened and endangered Chinook salmon and steelhead in the Sacramento–San Joaquin Basin (San Francisco Estuary Watershed Science, 2007), vol. 4. 10.15447/sfews.2007v5iss1art4.

29. R. S. Waples, Effective size of fluctuating salmon populations. *Genetics* **161**, 783–791 (2002).

30. T. F. Turner, M. J. Osborne, G. R. Moyer, M. A. Benavides, D. Alò, Life history and environmental variation interact to determine effective population to census size ratio. *Proc. R. Soc. B* **273**, 3065–3073 (2006).

31. R. S. Waples, Conservation genetics of Pacific salmon. III. Estimating effective population size. *J. Hered.* **81**, 277–289 (1990).

32. S. D. Voss, W. R. Poytress, 2021 Red Bluff diversion dam rotary screw trap juvenile anadromous fish abundance estimates. Report of US Fish and Wildlife Service to US Bureau of Reclamation, Sacramento, CA (2023).

33. A. M. Del Rio, G. N. Mukai, B. T. Martin, R. C. Johnson, N. A. Fangue, J. A. Israel, A. E. Todgham, Differential sensitivity to warming and hypoxia during development and long-term effects of developmental exposure in early life stage Chinook salmon. *Conserv. Physiol.* **9**, coab054 (2021).

34. D. E. Schindler, R. Hilborn, B. Chasco, C. P. Boatright, T. P. Quinn, L. A. Rogers, M. S. Webster, Population diversity and the portfolio effect in an exploited species. *Nature* **465**, 609–612 (2010).

35. R. Hilborn, T. P. Quinn, D. E. Schindler, D. E. Rogers, Biocomplexity and fisheries sustainability. *Proc. Natl. Acad. Sci. U.S.A.* **100**, 6564–6568 (2003).

36. R. Hallnan, L. Saito, D. Busby, S. Tyler, Modeling Shasta Reservoir water-temperature response to the 2015 drought and response under future climate change. *J. Water Resour. Plan. Manag.* **146**, 04020018 (2020).

37. T. N. Lipscomb, Z. Siders, S. Austing, J. V. Bargen, L. A. Earley, Accelerating the reintroduction of endangered Sacramento River winter-run Chinook Salmon to Battle Creek, California using captive broodstock. *N. Am. J. Fish. Manag.* **45**, 236–250 (2025).

38. National Marine Fisheries Service, Recovery plan for the evolutionarily significant units of Sacramento River winter-run Chinook salmon and Central Valley spring-run Chinook salmon and the distinct population segment of California Central Valley steelhead. *California Central Valley Area Office* (2014).

39. D. H. Secor, L. A. Kerr, S. X. Cadrin, Connectivity effects on productivity, stability, and persistence in a herring metapopulation model. *ICES J. Mar. Sci.* **66**, 1726–1732 (2009).

40. W. R. Poytress, W. T. McCraney, "2022 & 2023 Red Bluff Diversion Dam rotary trap juvenile anadromous fish abundance estimates" (Report of US Fish and Wildlife Service to US Bureau of Reclamation, Report of US Fish and Wildlife Service to US Bureau of Reclamation, Sacramento, CA, 2025).

41. A. M. Del Rio, B. E. Davis, N. A. Fangue, A. E. Todgham, Combined effects of warming and hypoxia on early life stage Chinook salmon physiology and development. *Conserv. Physiol.* **7**, coy078 (2019).

42. N. J. Mantua, H. Bell, A. E. Todgham, M. E. Daniels, J. Rinchard, J. M. Ludwig, J. C. Field, S. T. Lindley, F. E. Rowland, C. A. Richter, D. Walters, B. Finney, H. A. R. Distajo, D. Tillitt, D. C. Honeyfield, T. Lipscomb, K. Kwak, J. Kindopp, D. Cocherell, A. Ward, T. H. Williams, J. Harding, N. A. Fangue, C. Jeffres, R. I. Ruiz-Cooley, S. Y. Litvin, S. Foott, M. Adkison, B. Kormos, P. Harte, F. Colwell, C. P. Suffridge, K. C. Shannon, A. Cranford, C. Ambrose, A. Reed, R. C. Johnson, Widespread thiamine deficiency in California salmon linked to an anchovy-dominated marine prey base. *Proc. Natl. Acad. Sci. U.S.A.* **122**, e2426011122 (2025).

43. L. Bonacina, F. Fasano, V. Mezzanotte, R. Fornaroli, Effects of water temperature on freshwater macroinvertebrates: A systematic review. *Biol. Rev.* **98**, 191–221 (2023).

44. P. Pottier, S. Burke, R. Y. Zhang, D. W. A. Noble, L. E. Schwanz, S. M. Drobniak, S. Nakagawa, Developmental plasticity in thermal tolerance: Ontogenetic variation, persistence, and future directions. *Ecol. Lett.* **25**, 2245–2268 (2022).

45. V. Oborová, M. Šugerková, L. Gvoždík, Sensitivity of amphibian embryos to timing and magnitude of present and future thermal extremes. *J. Exp. Zool. A Ecol. Integr. Physiol.* **341**, 377–388 (2024).

46. L. G. Crozier, M. M. McClure, T. Beechie, S. J. Bograd, D. A. Boughton, M. Carr, T. D. Cooney, J. B. Dunham, C. M. Greene, M. A. Haltuch, E. L. Hazen, D. M. Holzer, D. D. Huff, R. C. Johnson, C. E. Jordan, I. C. Kaplan, S. T. Lindley, N. J. Mantua, P. B. Moyle, J. M. Myers, M. W. Nelson, B. C. Spence, L. A. Weitkamp, T. H. Williams, E. Willis-Norton, Climate vulnerability assessment for Pacific salmon and steelhead in the California Current Large Marine Ecosystem. *PLOS ONE* **14**, e0217711 (2019).

47. R. Barnett-Johnson, C. B. Grimes, C. F. Royer, C. J. Donohoe, Identifying the contribution of wild and hatchery Chinook salmon (*Oncorhynchus tshawytscha*) to the ocean fishery using otolith microstructure as natural tags. *Can. J. Fish. Aquat. Sci.* **64**, 1683–1692 (2007).

48. N. T. Kita, T. Ushikubo, B. Fu, J. W. Valley, High precision SIMS oxygen isotope analysis and the effect of sample topography. *Chem. Geol.* **264**, 43–57 (2009).

49. R. Kozdon, T. Ushikubo, N. T. Kita, M. Spicuzza, J. W. Valley, Intratest oxygen isotope variability in the planktonic foraminifer *N. pachyderma*: Real vs. apparent vital effects by ion microprobe. *Chem. Geol.* **258**, 327–337 (2009).

50. A. Pike, E. Danner, D. Boughton, F. Melton, R. Nemani, B. Rajagopalan, S. Lindley, Forecasting river temperatures in real time using a stochastic dynamics approach. *Water Resour. Res.* **49**, 5168–5182 (2013).

51. M. E. Daniels, V. K. Sridharan, E. M. Danner, S. N. John, Calibration and validation of linked water temperature models for the Shasta Reservoir and the Sacramento River from 2000 to 2015 (2018); 10.7289/V5/TM-SWFSC-597.

52. D. R. Geist, C. S. Abernethy, K. D. Hand, V. I. Cullinan, J. A. Chandler, P. A. Groves, Survival, development, and growth of fall Chinook salmon embryos, alevins, and fry exposed to variable thermal and dissolved oxygen regimes. *Trans. Am. Fish. Soc.* **135**, 1462–1477 (2006).

53. C. B. Murray, J. D. McPhail, Effect of incubation temperature on the development of five species of Pacific salmon (*Oncorhynchus*) embryos and alevins. *Can. J. Zool.* **66**, 266–273 (1988).

54. S. M. Blankenship, A. Scherer, C. Dean, K. Sellheim, J. Sweeney, J. Merz, Applying parentage methods to detect gravel augmentation effects on juvenile Chinook Salmon recruitment rates. *River Res. Appl.* **40**, 791–808 (2024).

55. A. R. Murdoch, T. N. Pearsons, T. W. Maitland, Use of carcass recovery data in evaluating the spawning distribution and timing of spring Chinook salmon in the Chiwawa River, Washington. *N. Am. J. Fish. Manag.* **29**, 1206–1213 (2009).

56. D. Killam, Sacramento River winter-run Chinook Salmon escapement survey April–September 2004. SRSSAP Technical Report No. 05–1 (2005).

57. J. C. Hegg, B. P. Kennedy, P. Chittaro, What did you say about my mother? The complexities of maternally derived chemical signatures in otoliths. *Can. J. Fish. Aquat. Sci.* **76**, 81–94 (2019).
